# Supplementary material for: Van der Waals nanomesh electronics on arbitrary surfaces
Source: Nat Commun. 2023 Apr 27;14:2431. doi: 10.1038/s41467-023-38090-8 (PMC10140039; doi:10.1038/s41467-023-38090-8)
Supplement: Supplementary file 1 — Supplementary Information [file 41467_2023_38090_MOESM1_ESM.pdf]

Supplementary Information

Van der Waals nanomesh electronics on arbitrary surfaces

You Meng[1,2,†], Xiaocui Li[1,3,†], Xiaolin Kang[1], Wanpeng Li[1], Wei Wang[1,2], Zhengxun Lai[1], Weijun Wang[1], Quan Quan[1], Xiuming Bu[1], SenPo Yip[4], Pengshan Xie[1], Dong Chen[1], Dengji Li[1], Fei Wang[5,*], Chi-Fung Yeung[6], Changyong Lan[7], Chuntai Liu[8], Lifan Shen[9], Yang Lu[3], Furong Chen[1], Chun-Yuen Wong[2,5,*], Johnny C. Ho[1,2,4,*]

[1] *Department of Materials Science and Engineering,* [2] *State Key Laboratory of Terahertz and Millimeter Waves, and* [3] *Department of Mechanical Engineering, City University of Hong Kong, Kowloon 999077, Hong Kong SAR*

[4] *Institute for Materials Chemistry and Engineering, Kyushu University, Fukuoka 816-8580, Japan*

[5] *State Key Laboratory of Luminescence and Applications, Changchun Institute of Optics, Fine Mechanics and Physics, Chinese Academy of Sciences, Changchun 130021, China*

[6] *Department of Chemistry, City University of Hong Kong, Kowloon 999077, Hong Kong SAR*

[7] *School of Optoelectronic Science and Engineering, University of Electronic Science and Technology of China, Chengdu 610054, P. R. China*

[8] *Key Laboratory of Advanced Materials Processing & Mold (Zhengzhou University), Ministry of Education, Zhengzhou 450002, P.R. China*

[9] *College of Microelectronics and Key Laboratory of Optoelectronics Technology, Faculty of Information Technology, Beijing University of Technology, Beijing 100124, P.R. China*

[†]*You Meng and Xiaocui Li contributed equally to this work.*

[*]*Corresponding author: Johnny C. Ho (johnnyho@cityu.edu.hk); Chun-Yuen Wong (acywong@cityu.edu.hk); Fei Wang (wangf@ciomp.ac.cn)*

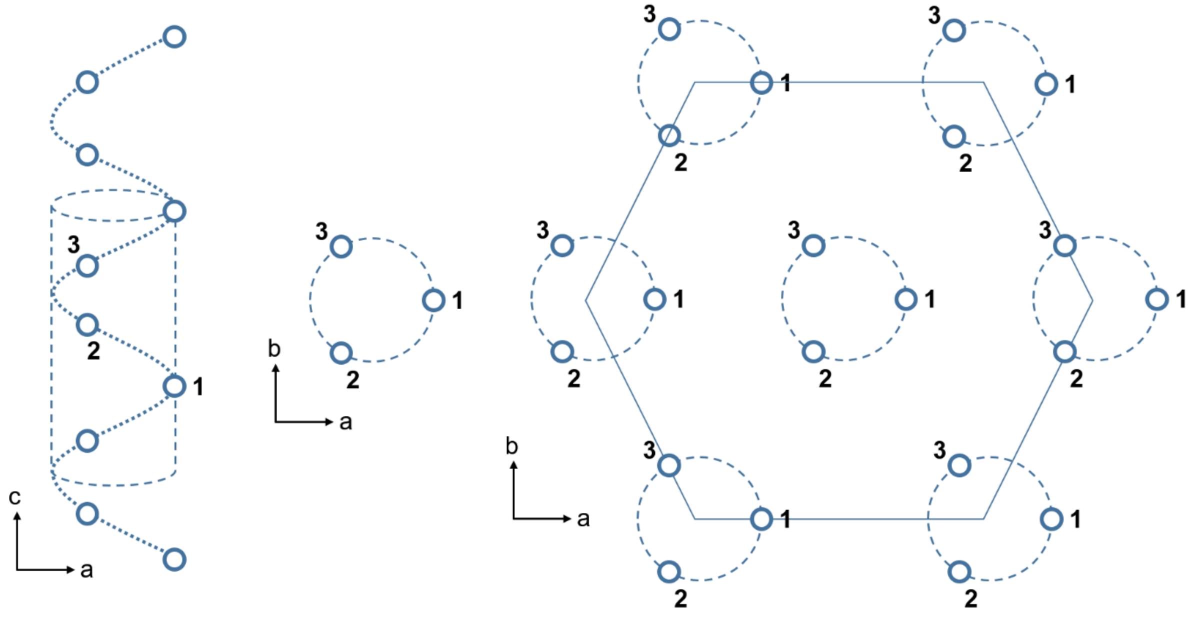

Supplementary Figure 1 Crystal structure of hexagonal Te materials. Spiral Te atomic chains are oriented along the c-axis, and held together by weak van der Waal forces to form a hexagonal crystal structure.

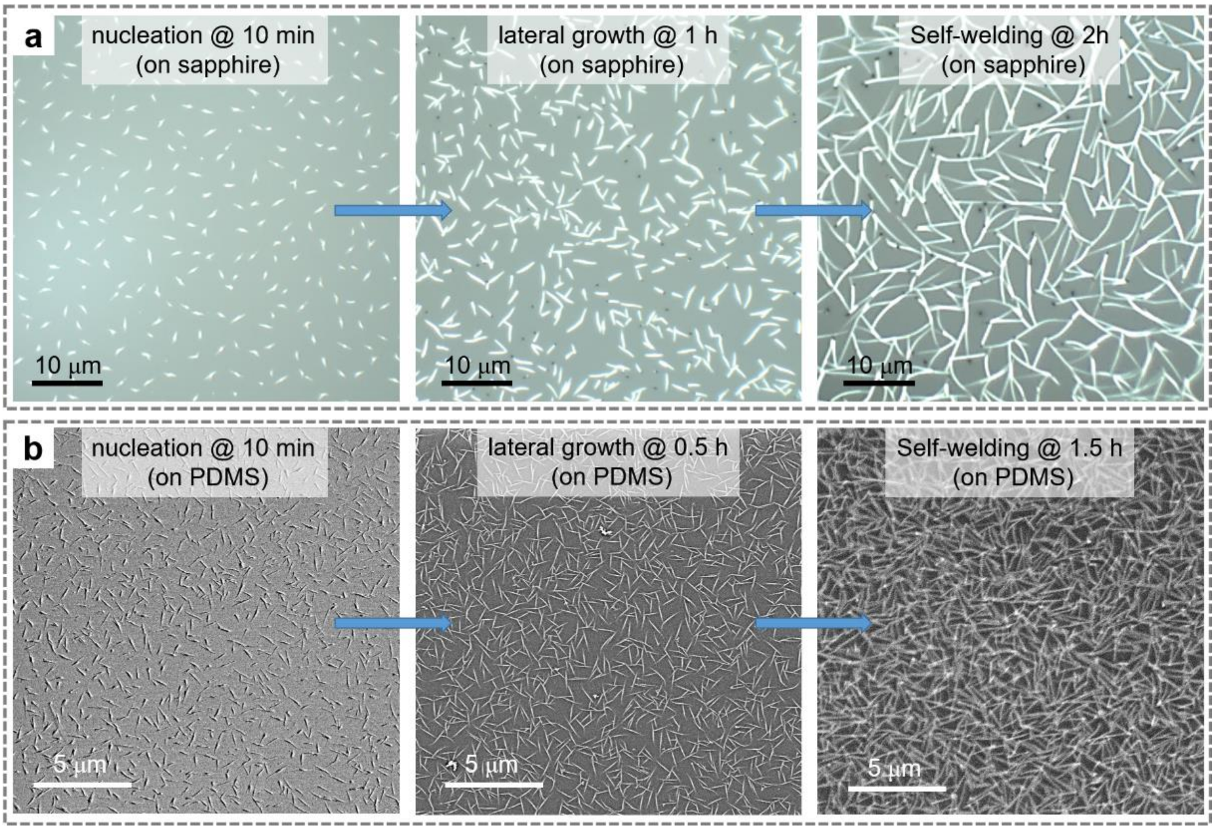

Supplementary Figure 2 (a) Optical images of Te nanomesh grown on sapphire with different growth durations. (b) SEM images of Te nanomesh grown on PDMS with different growth durations.

Supplementary Note 1

Although with distinctly different substrate features, Te nanomesh on both rigid and flexible substrates show basically similar growth behaviors. Taking rigid sapphire and flexible PDMS substrates as examples, where the nucleation, lateral growth and self-welding steps of Te NWs occur sequentially through the entire growth process. It is noted that the Te nanomesh grown on PDMS have the higher NW density (4.5 ± 0.2 μm^{-2}) than that of sapphire (0.45 ± 0.15 μm^{-2}), and thus decreasing the distance between two NWs. This would lead to the earlier occurrence of NWs contact and spontaneous self-welding process. For instance, with a short growth duration of 1~1.5 hours, the complete formation of self-welded nanomesh is observed on PDMS, being faster than the one of sapphire of 2~3 hours.

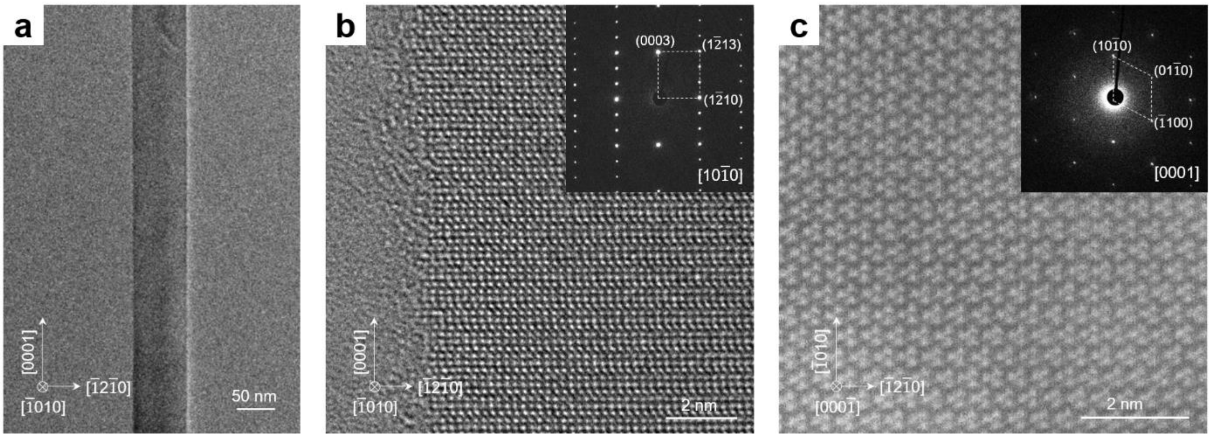

Supplementary Figure 3 (a) TEM image and (b) HRTEM image of a single-crystalline Te NW along $[10\bar{1}0]$ zone axis. (c) HAADF-STEM image of the cross-section of Te NW along c-axis $\langle 0001 \rangle$ direction. Insets show the corresponding SAED patterns. The favored c-axis $\langle 0001 \rangle$ growth of the Te NW is mainly because of the highly anisotropic crystalline structure of Te, being consistent well with the reported works[1,2].

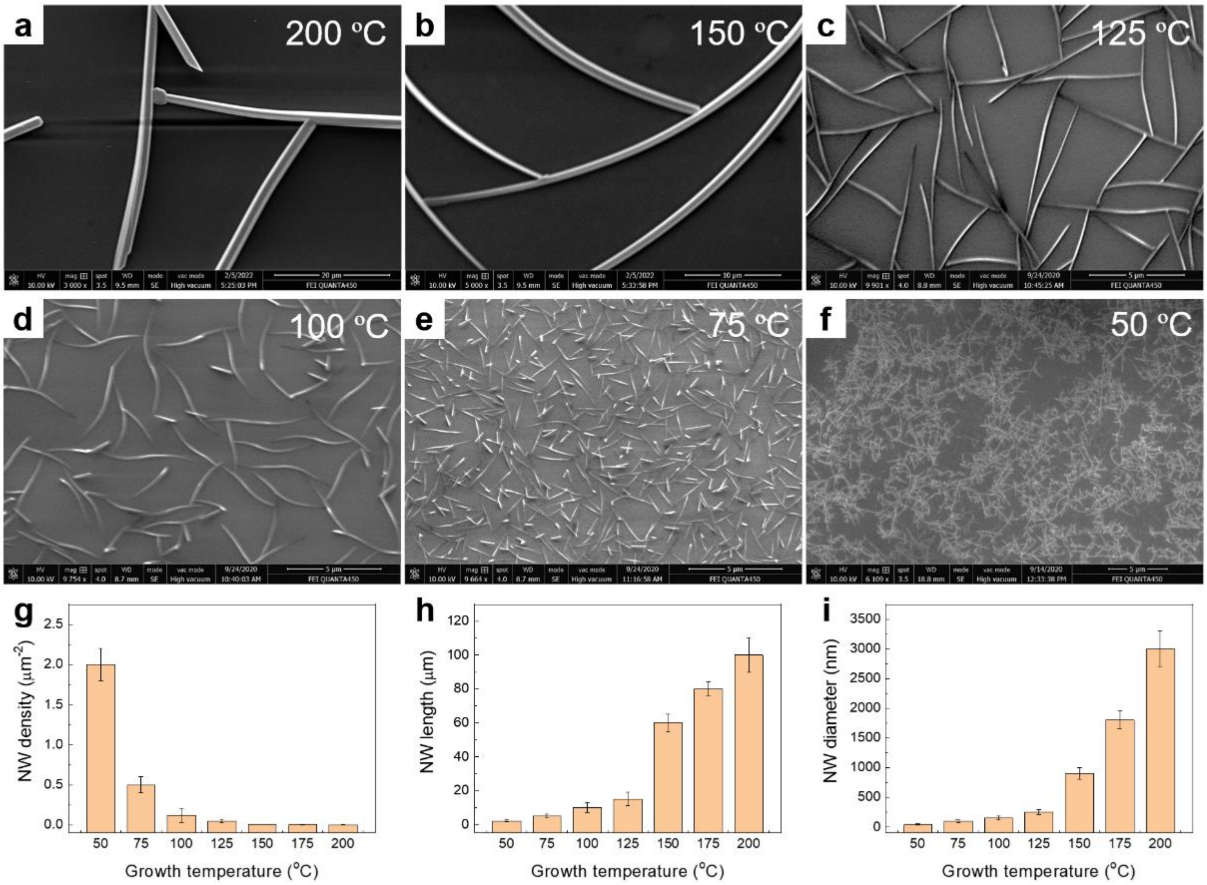

Supplementary Figure 4 SEM images of Te nanomesh grown on SiO_2 substrates with growth temperatures of (a) 200, (b) 150, (c) 125, (d) 100, (e) 75, and (f) 50 ºC. The corresponding (g) NW density, (h) NW length, and (i) NW diameter as a function of growth temperature.

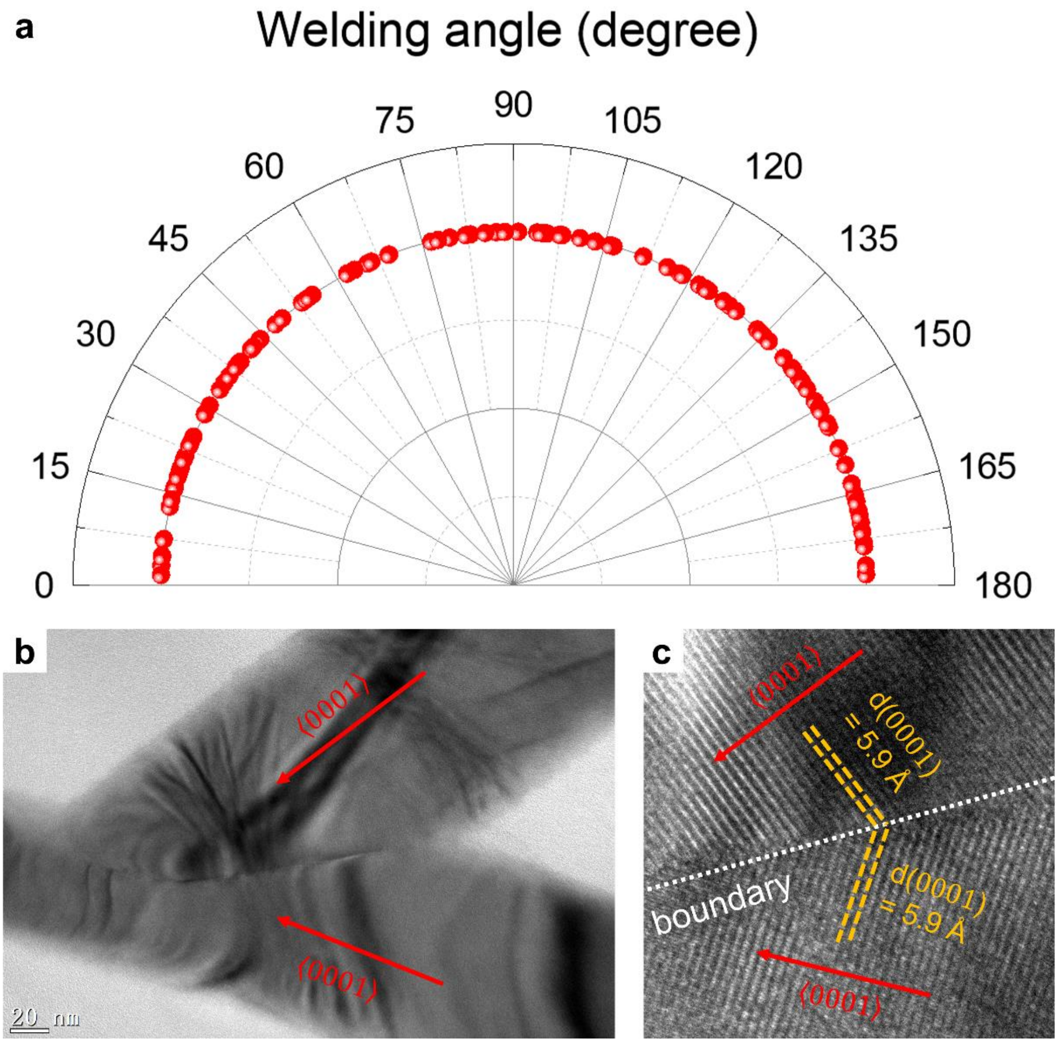

Supplementary Figure 5 (a) Welding angle distribution of Te nanomesh on sapphire. (b) TEM image of the NW welding region. (c) HRTEM image of the boundary between two welded NWs.

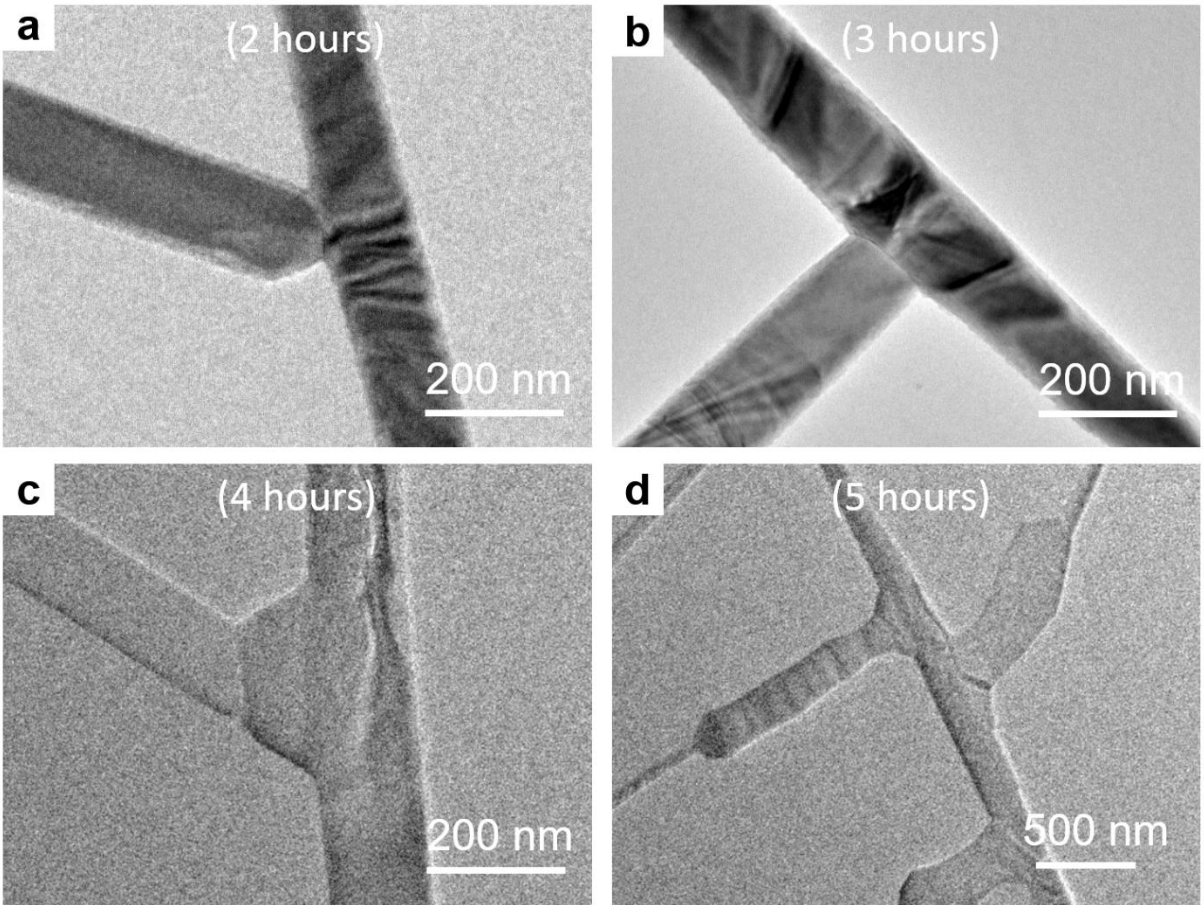

Supplementary Figure 6 TEM images of the self-welding process of Te nanomesh with different growth duration times of (a) 2 hours, (b) 3 hours, (c) 4 hours, and (d) 5 hours.

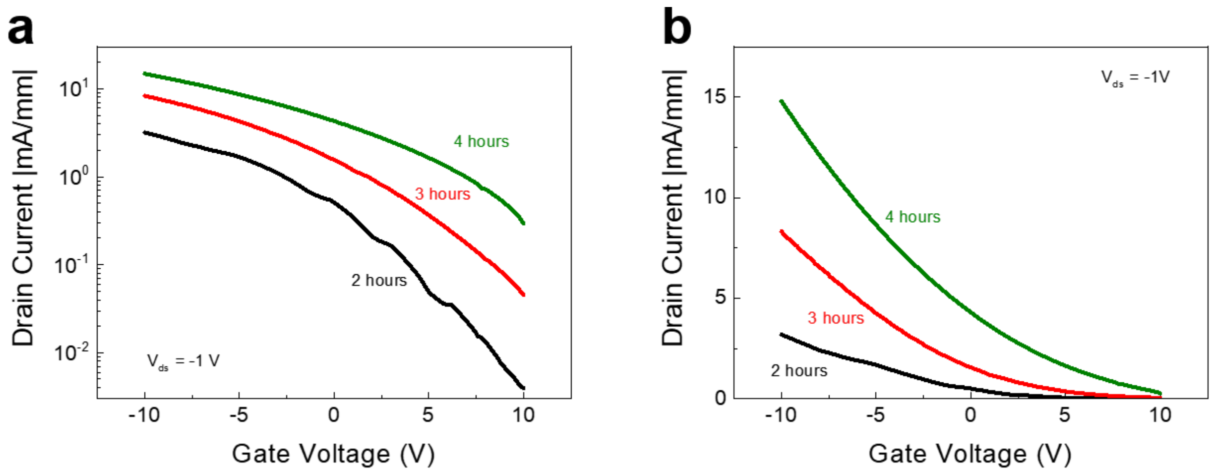

Supplementary Figure 7 Transfer curves of the Te nanomesh transistor with different growth duration times using (a) logarithm y-coordinate and (b) linear y-coordinate. The reinforced welding points in nanomesh with longer growth duration would enhance the carrier transport ability of nanomesh. For instance, with growth time increasing from 2, 3, to 4 hours, the hole mobilities of nanomesh increased from 108, 145, to 181 cm^2/Vs, respectively. This adds an addition dimension to tune the electrical properties of p-type Te nanomesh.

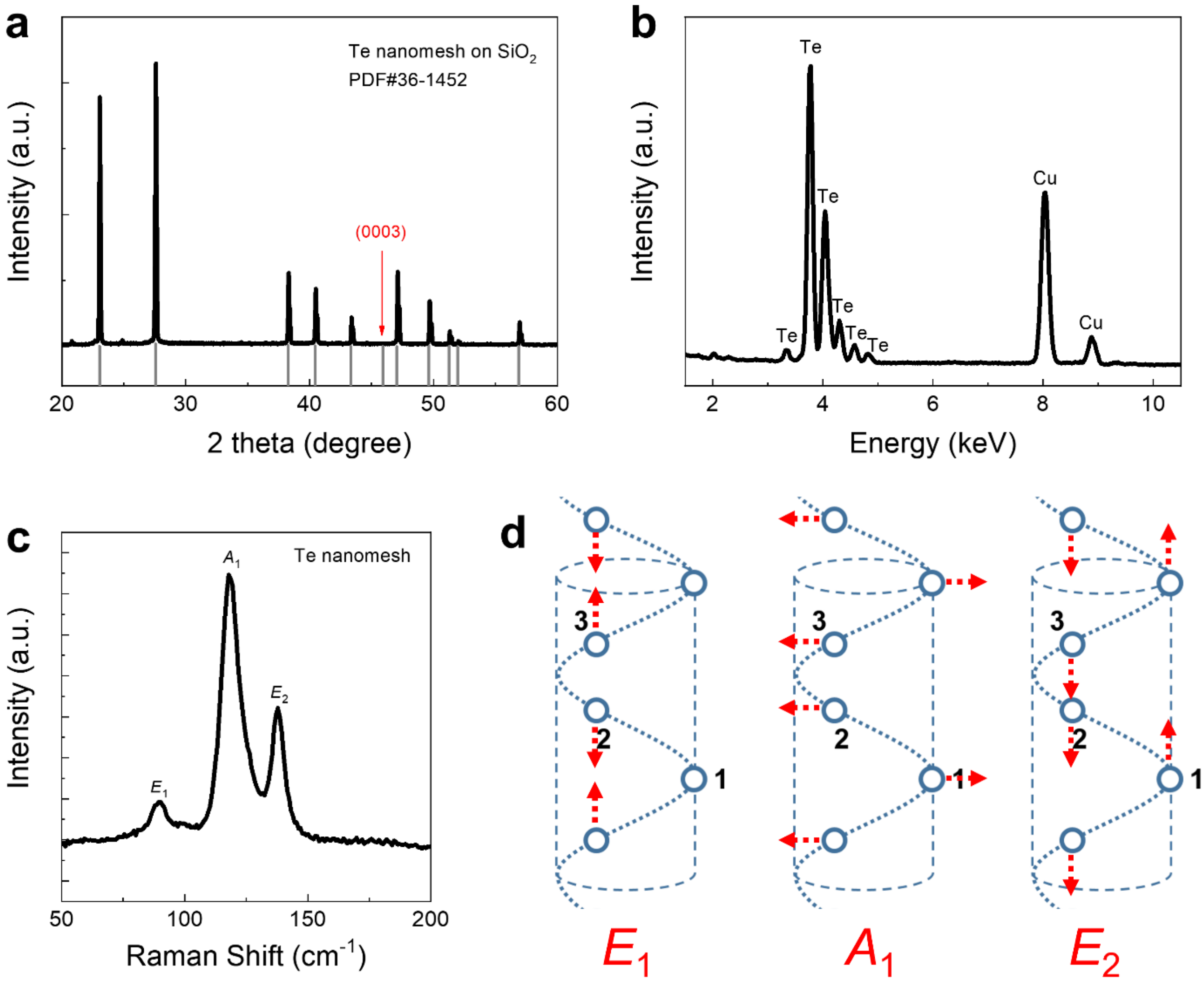

Supplementary Figure 8 (a) XRD patterns of Te nanomesh grown on 270-nm SiO_2/p^{++} Si substrates. All the diffraction peaks are in perfect agreement with the typical Te hexagonal crystal (JCPDS card, no. 36-1452, $P3_121$ [152]). Because the packed Te molecular chains are in the plane of the growth substrates, the (0003) diffraction peak is of absence in the XRD pattern. (b) EDS analysis of Te nanomesh, which shows the compositional purity. (c) Raman studies of Te nanomesh, where three first-order Raman active modes located at 89.7 cm^{-1} (E_1 transverse (TO) phonon mode), 118.1 cm^{-1} (A_1 mode) and 137.8 cm^{-1} (E_2 mode) were identified. (d) Schematic vibration patterns of the Raman modes of E_1, A_1, and E_2 in chiral-chain Te materials[1]. The E_1 and E_2 modes represent bond-bending and bond-stretching with a larger admixture, respectively, whereas the A_1 mode is caused by chain expansion in the basal plane.

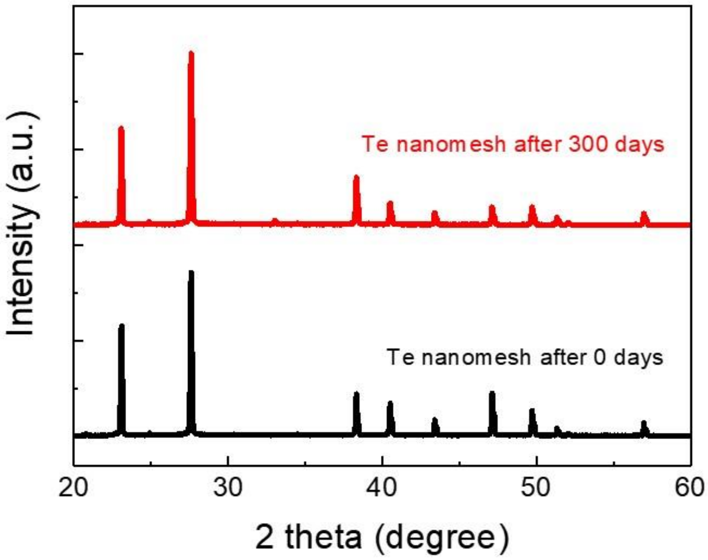

Supplementary Figure 9 XRD patterns of Te nanomesh measured as fabricated and after 300-days storage in ambient conditions. After stored in ambient conditions for 300 days, the hexagonal-phase diffraction peaks are unaltered in terms of both peak intensity and peak position, highlighting the superior environmental stability of Te nanomesh against environmental factors, such as moisture and atmospheric oxygen. Also, the absence of the (0003) peak indicates that Te molecular chains are aligned in the plane of the substrate.

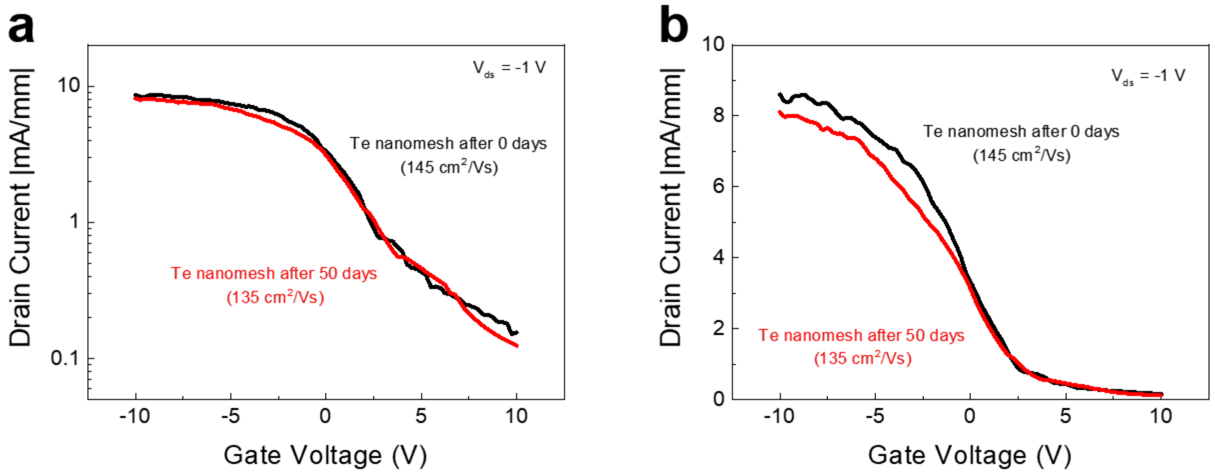

Supplementary Figure 10 Transfer curves of the Te nanomesh transistor measured as fabricated and after 50-days storage in ambient conditions using (a) logarithm y-coordinate and (b) linear y coordinate. Benefiting from the excellent material durability of Te nanomesh, the devices exhibited stable operational stability under the long-term storage test. To our best knowledge, the superior environmental durability of Te nanomesh is unachievable by other p-type counterparts.

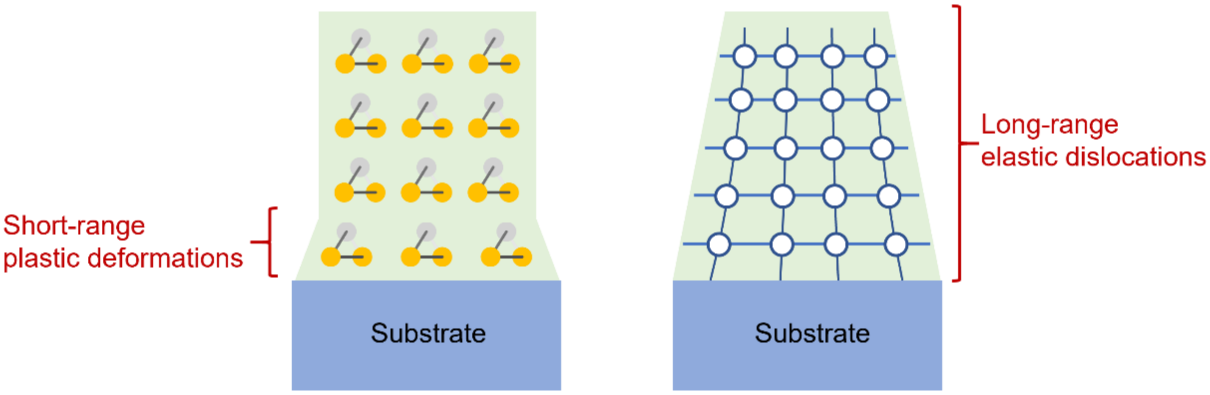

Supplementary Figure 11 Strain relaxation schematics of (left) short-range plastic deformations and (right) long-range elastic dislocations.

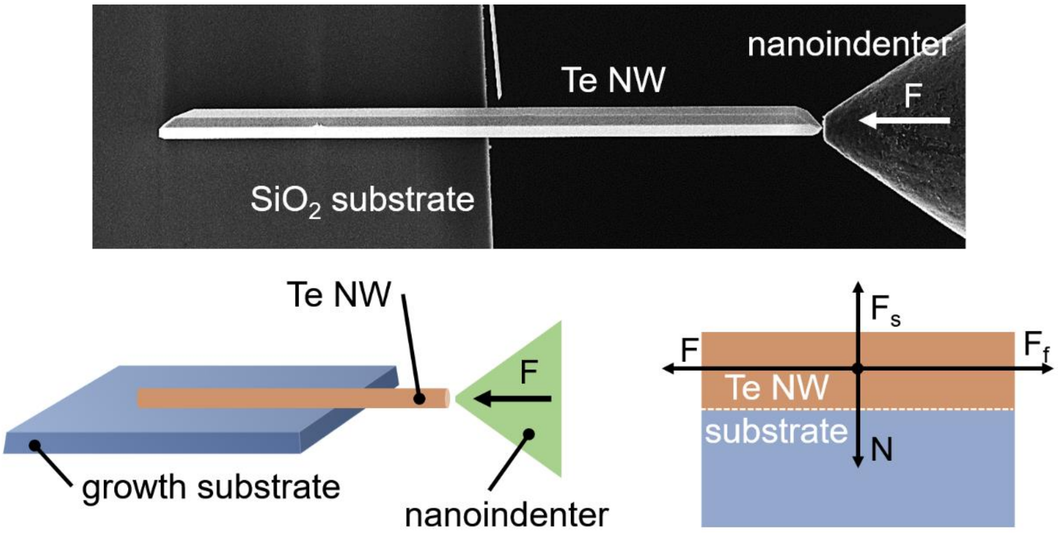

Supplementary Figure 12 SEM image and schematic illustration of the friction property test of Te NW on growth substrate.

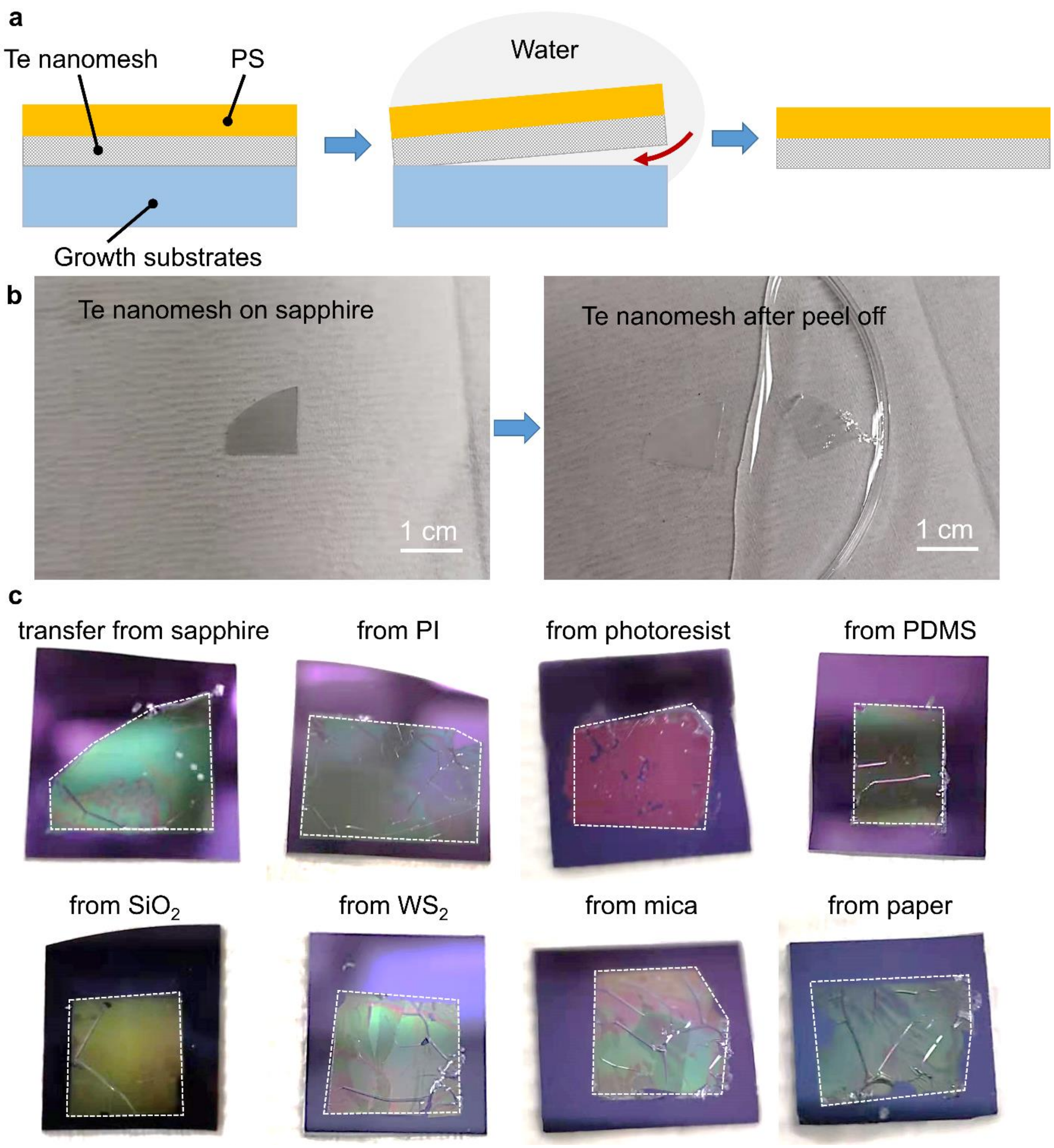

Supplementary Figure 13 (a) Schematic illustration of the surface-energy-assisted transfer process of Te nanomesh. (b) Photographs of Te nanomesh on sapphire growth substrate and after peel off in water. (c) Photographs of the peeled-off nanomesh from various donor substrates transferred onto target substrates.

Supplementary Note 2

A typical surface-energy-assisted process,[3] widely used to transfer vdWs layered materials previously, is employed here to peel off centimeter-scale Te nanomesh from the original growth substrates (Supplementary Figure 13a). Here, polystyrene (PS) polymer layer was spin-coated on top of the as-grown Te nanomesh to act as carrier polymers that help with the handling of the ultrathin nanomesh. Enabled by the surface energy difference and weak van der Waals

interaction between Te nanomesh and growth substrate, the water penetration into nanomesh/substrate interface provides a mild peel-off process of Te nanomesh from substrates (Supplementary Figure 13b). As a result, a complete peel-off of the nanomesh materials from various substrates and then a complete release of the peeled-off nanomesh onto target substrates were demonstrated in Supplementary Figure 13c. Such a room-temperature etching-free peel-off process is promising to transfer nanomesh for further utilizations and, more importantly, also highlights the quasi van der Waals interaction between Te nanomesh and growth substrates.

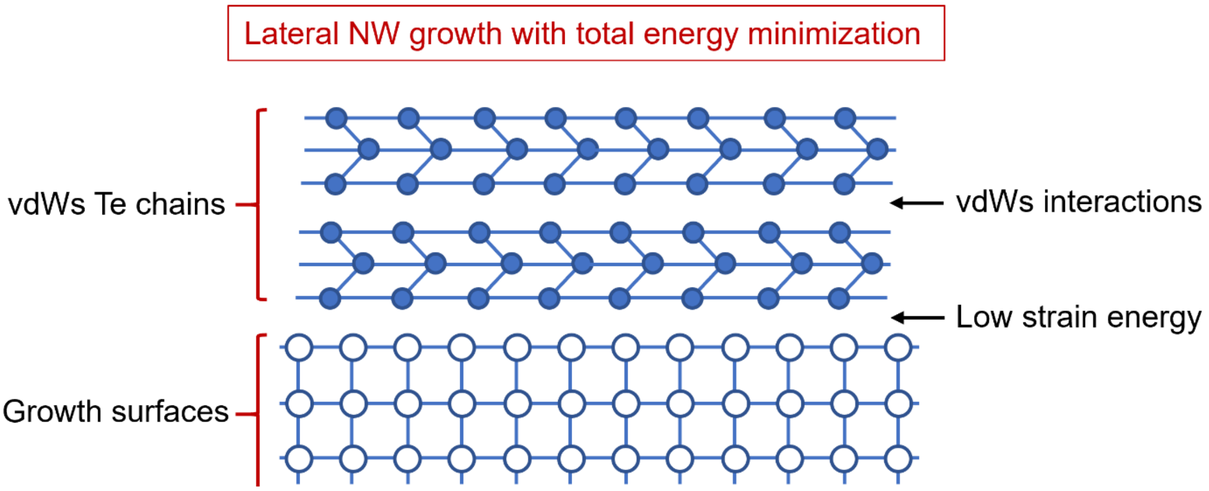

Supplementary Figure 14 Schematic diagram of the lateral Te NW growth with total energy minimization. Following the typical thermodynamic model, the energy minimizations could determine the lateral growth of Te NWs on substrates.[4,5] The covalently-bonded Te atoms along the c-axis have higher binding energy (0.68 eV) than those along the $\langle\bar{1}2\bar{1}0\rangle$ (0.22 eV) and $\langle 10\bar{1}0\rangle$ (0.05 eV) directions. Therefore, in a continuous adsorption and desorption process, driven by the stronger bond in $\langle 0001\rangle$ directions, the growth along the c-axis (i.e., the NW axial direction) is energetically favored. On the other hand, as the STEM results shown in Fig. 3a (main text), the strain energy between Te and substrate is efficiently released by the short-range plastic deformations that happened parallel to the substrate. Overall, the Te NWs would grow laterally on substrates because the corresponding energy of the whole system is minimized.

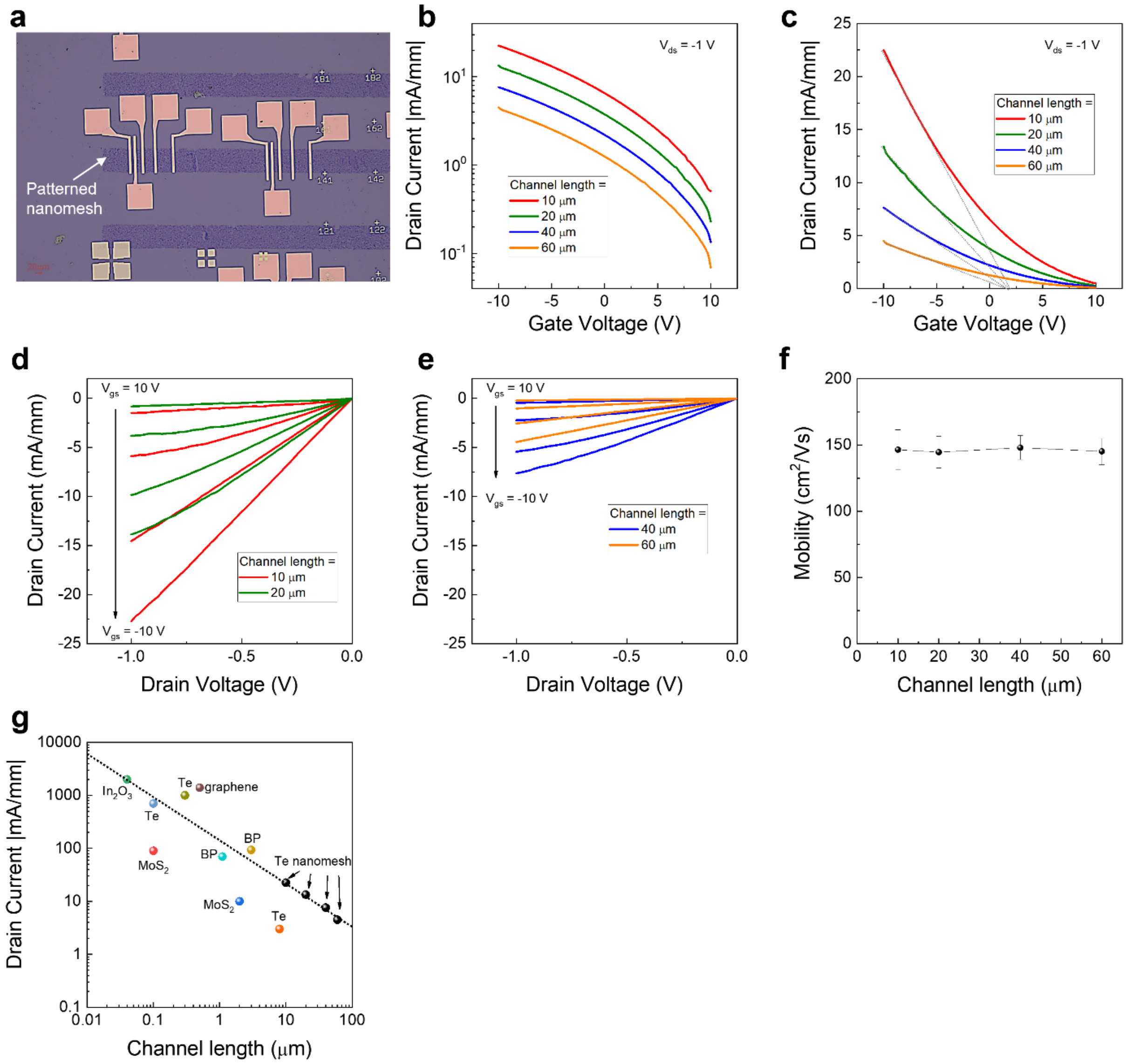

Supplementary Figure 15 (a) Device image of the electrical measurement performed in this work. (b, c) Transfer curves, (d, e) output curves, and (f) corresponding hole field-effect mobilities of the Te nanomesh transistors with different channel lengths. (g) Drain current densities of Te nanomesh and some typical semiconductors, including Te,[1,6,7] black phosphorus (BP),[8,9] In_2O_3,[10] and MoS_2.[11]

Supplementary Note 3

Te nanomesh transistors with different channel lengths of 10, 20, 40, and 60 μm were fabricated for the transfer length method (TLM) measurement. Typical transfer curves together with similar hole field-effect mobilities around 145 cm^2/Vs are obtained at different channel lengths, indicating a good uniformity of Te nanomesh. In order to clearly show the current-carrying capacity, we summarize the drain current densities of Te nanomesh and some typical semiconductors, including Te, BP, In_2O_3, and MoS_2. As shown in Supplementary Figure 15g,

the good current-carrying capacity of Te nanomesh is highlighted with different channel lengths. This way, the potentially high current density comparable to those of typical semiconductors is possibly obtained in a short channel device, but only if the contact resistance and boundary resistance could be optimized.

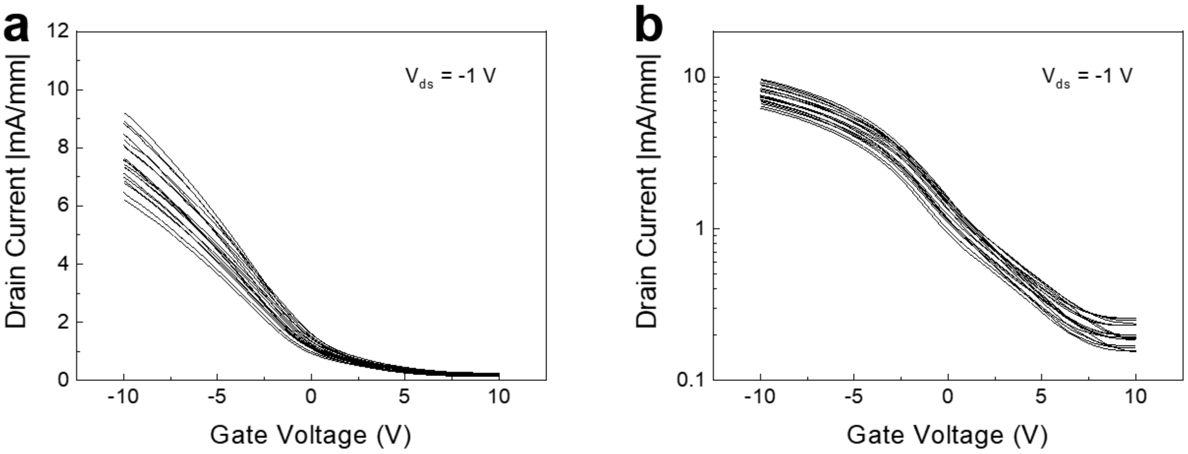

Supplementary Figure 16 Transfer curves of the 20 Te nanomesh TFTs in a 4 × 5 array from the same wafer, using the (a) linear y-axis and (b) logarithmic y-axis. After the statistical analysis of 20 devices, the on-current of 7.5 ± 1.0 mA/mm, the threshold voltage of 1.8 ± 0.3 V, and hole mobility of 145 ± 15 cm^2/Vs are obtained, proving the excellent uniformity of nanomesh TFT performance.

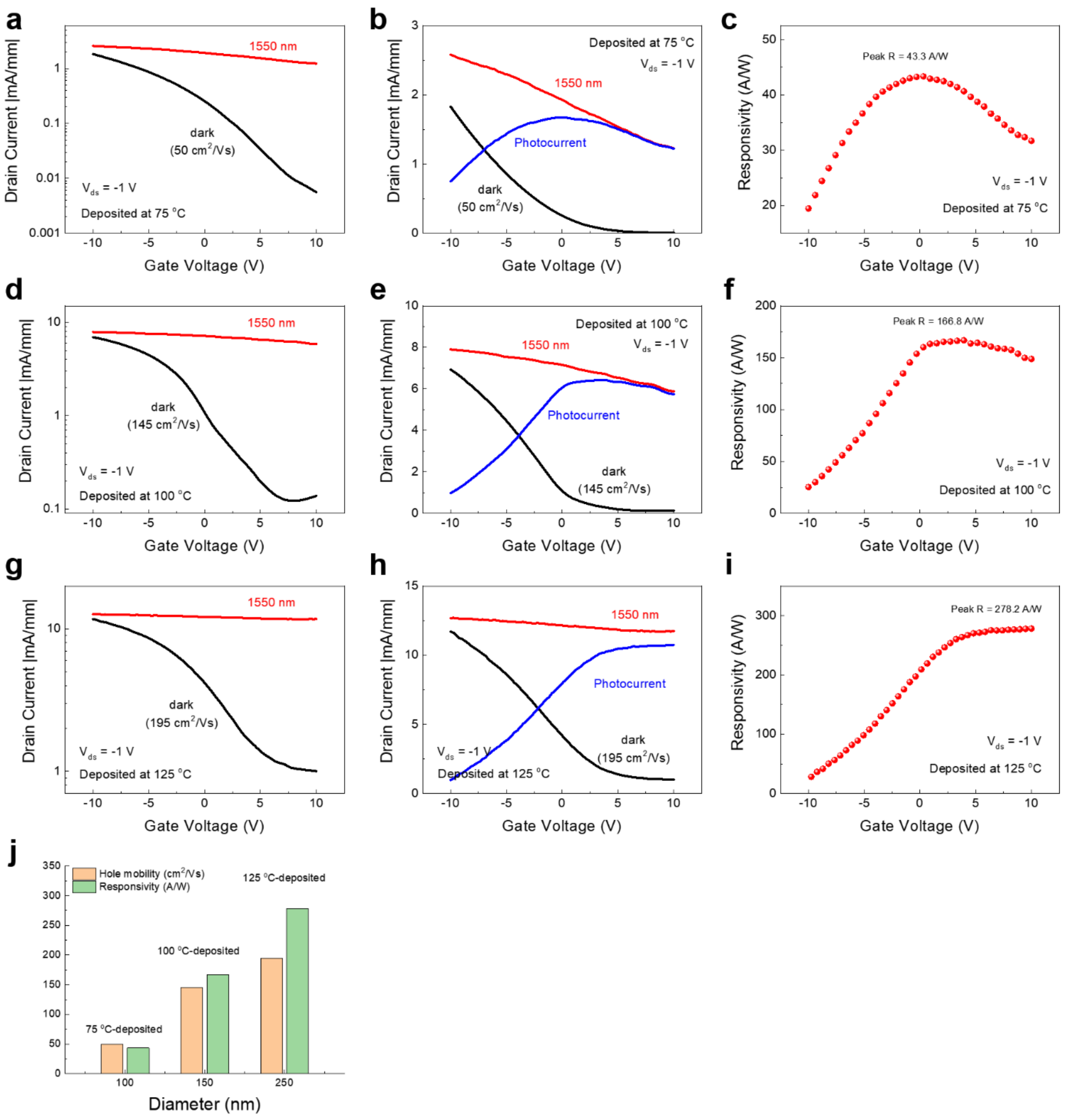

Supplementary Figure 17 Phototransistor performances of the Te nanomesh/SiO_2 substrate deposited at different temperatures of (a-c) 75 ºC, (d-f) 100 ºC, and (g-i) 125 ºC. (j) The relationship between the thickness and the electrical performance of nanomesh.

Supplementary Note 4

Indeed, the morphology and dimension of Te nanomesh affect their device performance. Here, we measure the phototransistor performance of Te nanomesh with different deposition temperatures, i.e. different morphologies. As shown in Supplementary Figure 17j, with the larger diameters of Te NW and fewer boundaries among Te nanomesh, the nanomesh deposited at higher temperatures show the better hole mobility and higher light responsivity. For instance, the 250 nm-diameter (125 ºC-deposited) Te nanomesh have a hole mobility of 195 cm^2/Vs, being higher than those of 150 nm-diameter (100 ºC-deposited) Te nanomesh of 145 cm^2/Vs and 100 nm-diameter (75 ºC-deposited) Te nanomesh of 50 cm^2/Vs, respectively. Also, it is found that the photoresponse can be strongly modulated by the nanomesh diameter control. To be specific, for devices grown with the diameter of 100, 150, and 250 nm, the corresponding peak responsivities of ~ 43.3, 166.8, and 278.2 A/W are observed, accordingly.

Supplementary Note 5

There are several reasons for the high on/off current ratio and good photo-gating effect of Te nanomesh. First, the unique out-of-plane van der Waals force of Te materials makes them free from the surface dangling bonds compared to traditional materials, which reduces the dark current of the devices from surface recombination. Second, because of the high surface-to-volume ratio and optical cavity effect, the strong light-matter interaction of one-dimensional materials enables them to exhibit excellent photoelectric conversion. Third, it is mainly as a result from the intrinsic high hole mobility of Te nanomesh, providing efficient collection/transport ability of photo-generated carriers. Finally, the Te nanomesh explored in this work are composed of many single-crystal Te NWs and their well-connected crystal boundaries at self-welding positions. Those crystal boundaries can form Schottky barriers to impede the motion of free carriers from one NW to another, which suppresses the off current as well as enhance the photo-gating effect.

Supplementary Note 6

In this work, the fabricated Te nanomesh devices show improved field-effect hole mobility in some cases with different material systems, channel structures, etc. The reasons for the altering mobility when compared with different types of channel counterparts are discussed from the viewpoints of Te material property, self-welding process, and nanomesh geometry.

First, comparing to scalable p-type semiconducting thin films, the obtained hole mobility value (~145 cm^2/Vs) of Te nanomesh in this work outperforms most of the p-type polycrystalline thin films, including metal oxides, metal halides, perovskites, organic materials, and evaporated Te-based films (detailed performance parameters can be found in Supplementary Table 3). The intrinsically high hole mobility of Te materials up to thousands cm^2/Vs, few crystal defects of individual Te NW among the nanomesh, and relatively few grain boundaries in the channel are the main reasons for the higher hole mobility of the nanomesh as compared to the conventional p-type polycrystalline semiconducting thin films. In this work, all the morphological and crystallographic features of the Te nanomesh are evaluated in detail

and confirmed by SEM, TEM, and HAADF-STEM studies. These results show that the material properties of Te nanomesh could be well controlled, enabling effective modulation and improving the device performance, including the field-effect hole mobility.

Second, the obtained mobility is higher than those of NW networks or metal oxide nanofiber networks that usually possess field-effect mobility values of 1~10 cm^2/Vs. Our results and the reported literature show that the self-welding process of NW networks or nanomesh is essential for promoting their performance in electrical devices. Compared to the NW networks formed by solution-phase deposition schemes that always give the weak physical inter-NW connection, the self-welded nanomesh with well-connected network morphology is expected to lower the contact barrier and reduce inter-NW junction resistance, making them a potentially high-performance device channel. To better show the nanomesh growth control, self-welding process, and corresponding device performance optimization of Te nanomesh, the related experiments were carried out as a function of different growth durations (Supplementary Figure 7) and deposition temperatures (Supplementary Figure 17). With the tunable diameter, length, and welding status of the Te nanomesh, we could obtain a platform to achieve hole mobility of ~145 cm^2/Vs in a controllable and reliable way.

Third, we would like to note that the obtained hole mobility values of Te nanomesh in this work are still significantly lower than some single-crystal Te nanostructures, like solution-grown 2D Te layers of 700 cm^2/Vs,[7] 1D Te NW encapsulated in a nanotube of 600 cm^2/Vs,[1] and single Te NW up to 1390 cm^2/Vs,[12] which is reasonable when considering the wafer-scale coverage and inter-NW connections among nanomesh. Also, the theoretical studies proposed that the Te materials have potentially high hole mobility up to ~10^5 cm^2/Vs and small carrier effective mass along the y direction.[13] Thus, there is still ample spacing to further improve the mobility of Te nanomesh with various strategies in the future.

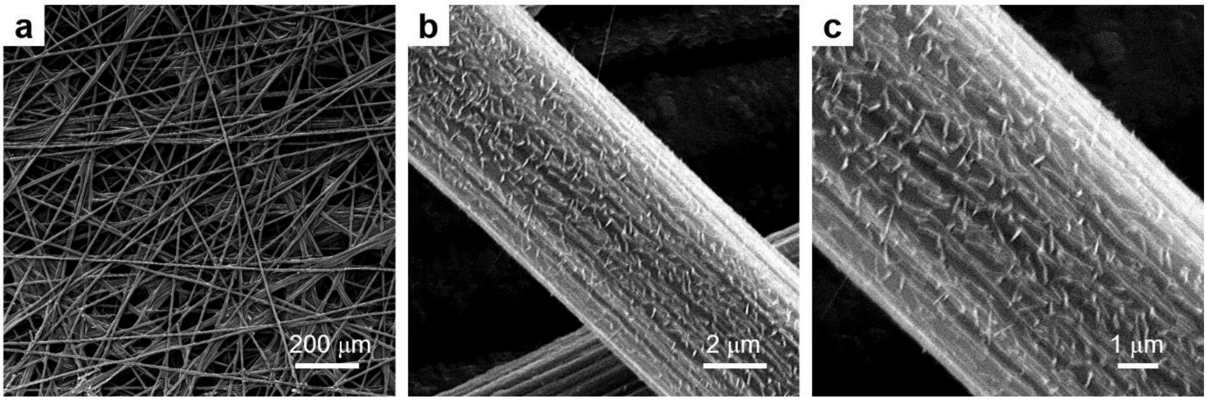

Supplementary Figure 18 (a-c) SEM images on Te nanomesh grown on carbon fibers with a growth duration of 3 hours. The unique capability of multi-substrate compatible growth is capable of growing the Te nanomesh on curved substrates, such as on carbon fibers. This is difficult to be achieved by conventional contact printing or solution-processed NW networks, as these methods rely heavily on mechanical assembly or solution-phase shear force.

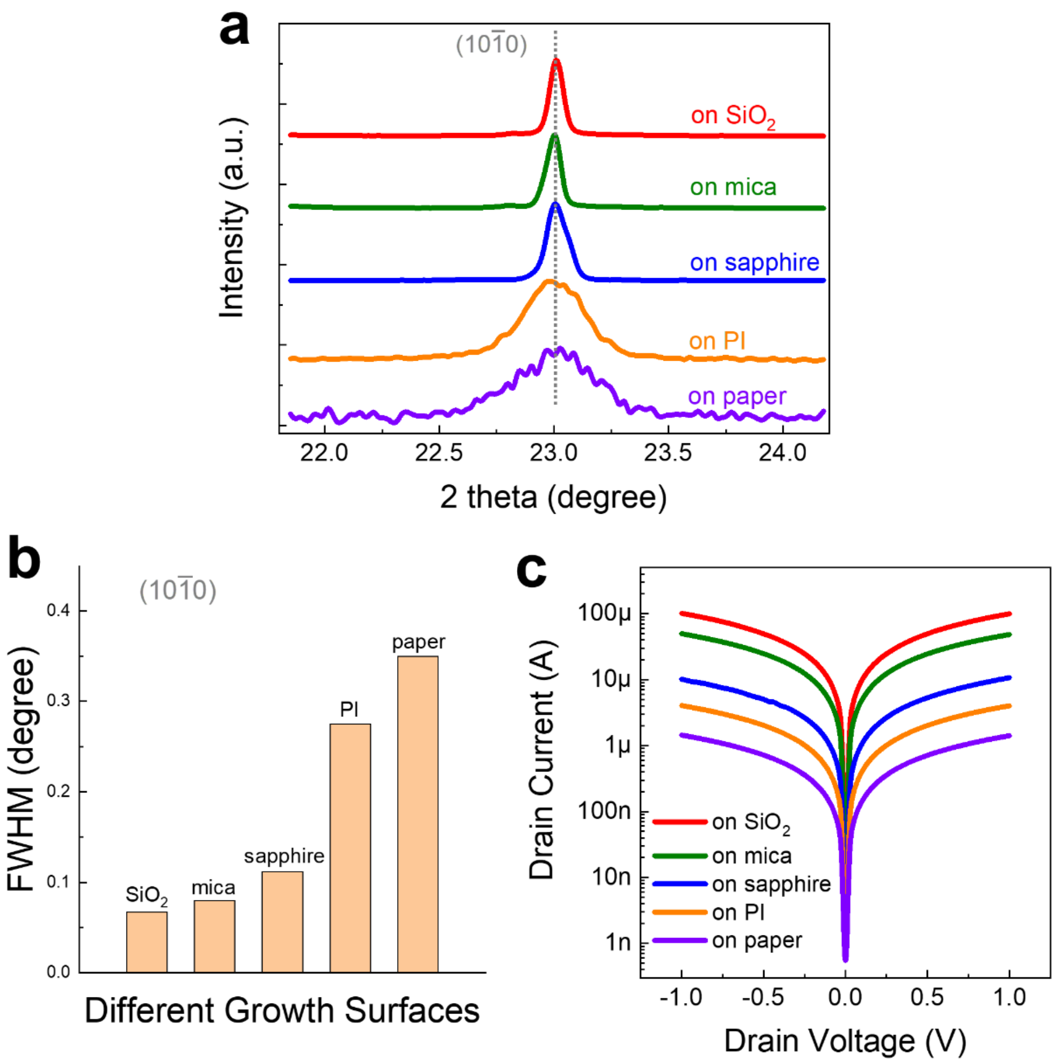

Supplementary Figure 19 (a) XRD spectra of $(10\bar{1}0)$ peak and (b) corresponding FWHM values of Te nanomesh grown on different substrates. (c) I-V curves of Te nanomesh grown on different substrates.

Supplementary Note 7

The morphologies of Te nanomesh grown on various substrates are different in term of rate, diameter, length, and density. This is mainly because the densities of active surface sites (steps, defects, impurities, etc.) depend on the substrate type and substrate quality. As for the crystallinity and electrical properties of the obtained Te nanomesh on different substrates, we measured their XRD patterns and I-V curves. With growth substrates changing from SiO_2, mica, sapphire, polyimide to paper, the XRD diffraction peaks of the Te nanomesh were gradually broadened, in which the full width at half maximum (FWHM) of $(10\bar{1}0)$ plane changes from 0.07, 0.08, 0.11, 0.28, to 0.40 °, respectively. This means the decreased average grain sizes and increased NW boundaries within the nanomesh, which are related to the densities of active surface sites of different substrates. At the same time, their peak positions are always located at ~ 30 °, which means their host lattice structure does not experience substitutional contraction or expansion. In general, the material crystallinity has a critical influence on its electrical

properties. Here, with different substrates, the electrical conductivities of Te nanomesh witness a decreasing trend from SiO_2, mica, sapphire, polyimide to paper. Overall, the decreased grain boundaries within the Te nanomesh would generate better electrical conductivities.

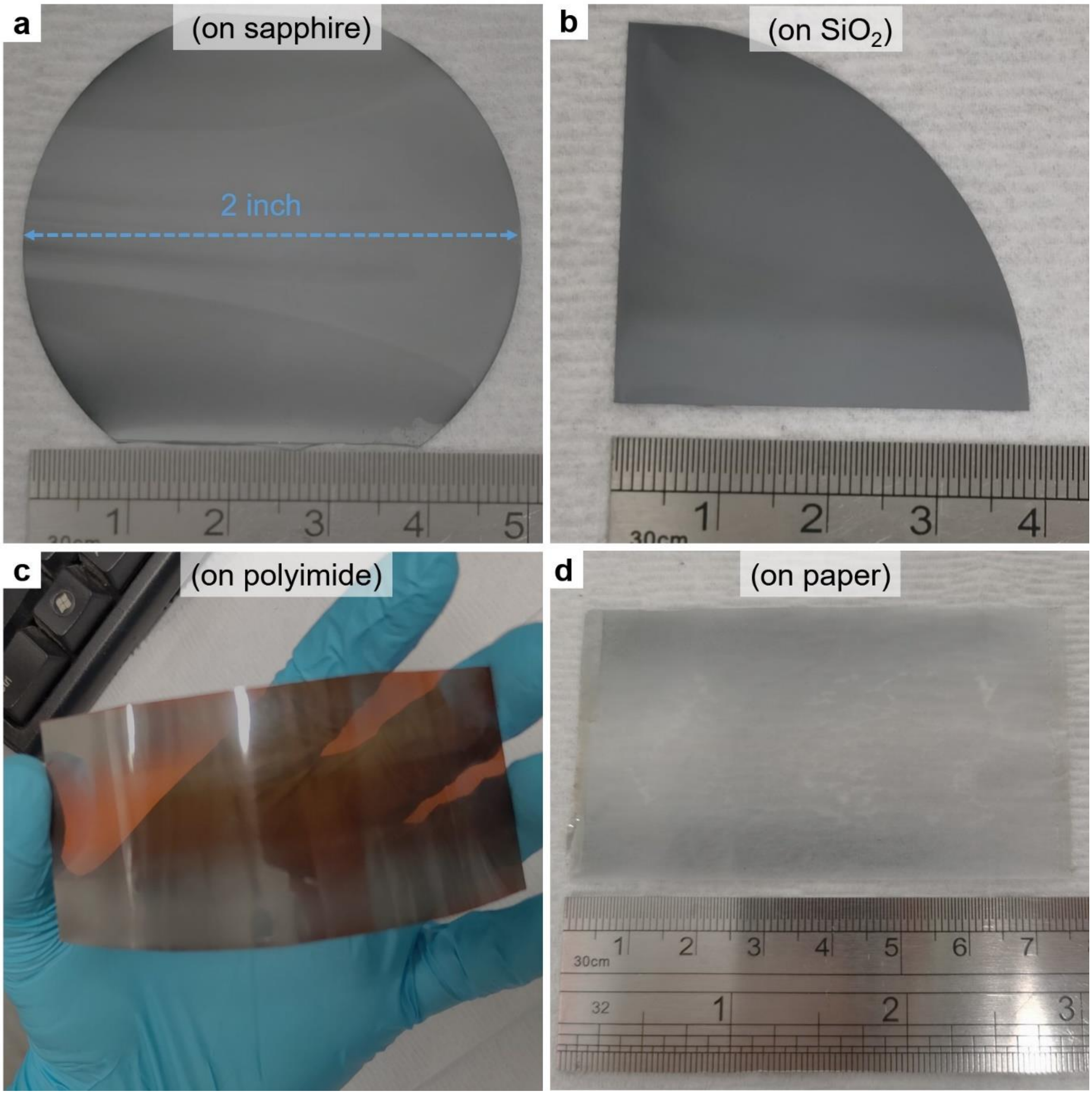

Supplementary Figure 20 Photographs of wafer-scale growth of Te nanomesh on (a) sapphire, (b) SiO_2, (c) PI, and (d) weighing paper. The wafer-scale growth is carried out by equipping the vapor transport system with a 2-inch quartz tube. As a result, wafer-scale Te nanomesh is uniformly yielded on different kinds of substrates.

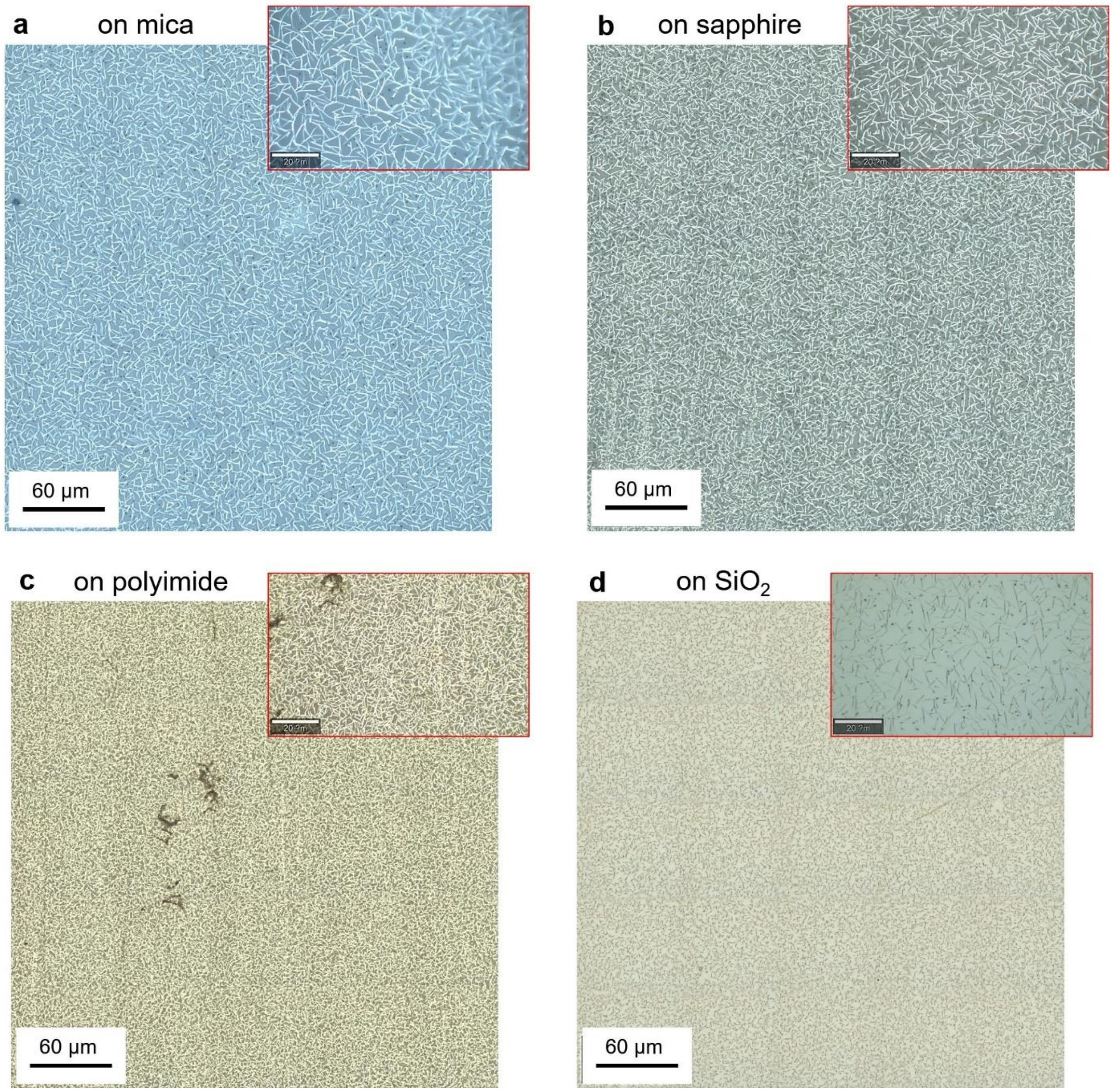

Supplementary Figure 21 Optical images of wafer-scale growth of Te nanomesh on (a) mica, (b) sapphire, (c) PI, and (d) SiO_2. All the substrates are fully covered by the well-connected nanomesh after growth, further highlighting the feasibility of Te nanomesh methodology.

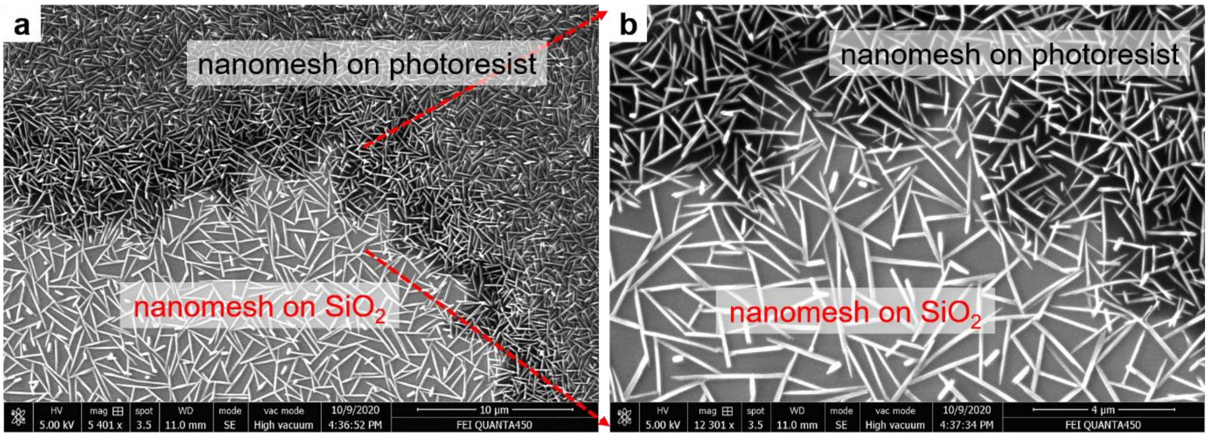

Supplementary Figure 22 (a, b) SEM images of Te nanomesh grown on both the pre-patterned photoresist film and the uncovered SiO_2/p^{++} Si substrates. Taking advantage of the multi-substrate compatible growth, Te nanomesh were grown on both the pre-patterned photoresist film and the photoresist-uncovered SiO_2/p^{++} Si substrates simultaneously. Also, it should be noted that the NW density of Te nanomesh on the photoresist-uncovered SiO_2/p^{++} Si substrates (1.7 μm^{-2}) is distinctively higher than that of pristine SiO_2/p^{++} Si substrates (0.1 μm^{-2}) that not experienced exposing/removing process. This is mainly because the residual chemicals on photoresist-uncovered SiO_2/p^{++} Si substrates that resulted from the lithography process act as active surface sites to nucleate the Te NW growth. Thus, manipulating the active surface sites (steps, defects, impurities, etc.) through controllable ways, e.g., chemical treatment and plasma treatment, could be reliable routes to alter the NW density of Te nanomesh in the future.

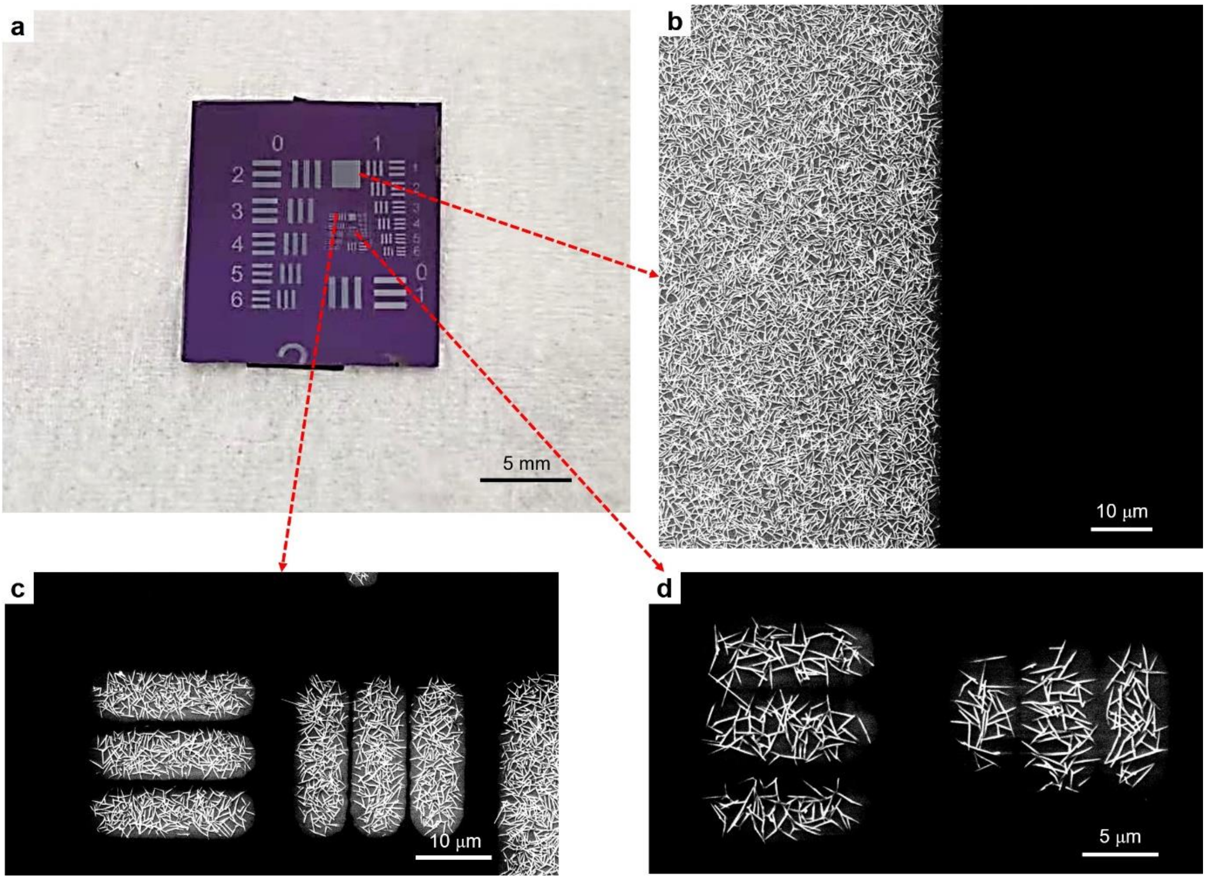

Supplementary Figure 23 (a) Photograph of patterned nanomesh in USAF 1951 resolution test. (b) and (c) SEM images of patterned images, showing high resolutions and distinct edges. (d) The finest pattern (the 6st element in the 6st group) obtained in USAF 1951 resolution test, corresponding to a resolution of 228.1 line pairs per millimeter (lp/mm).

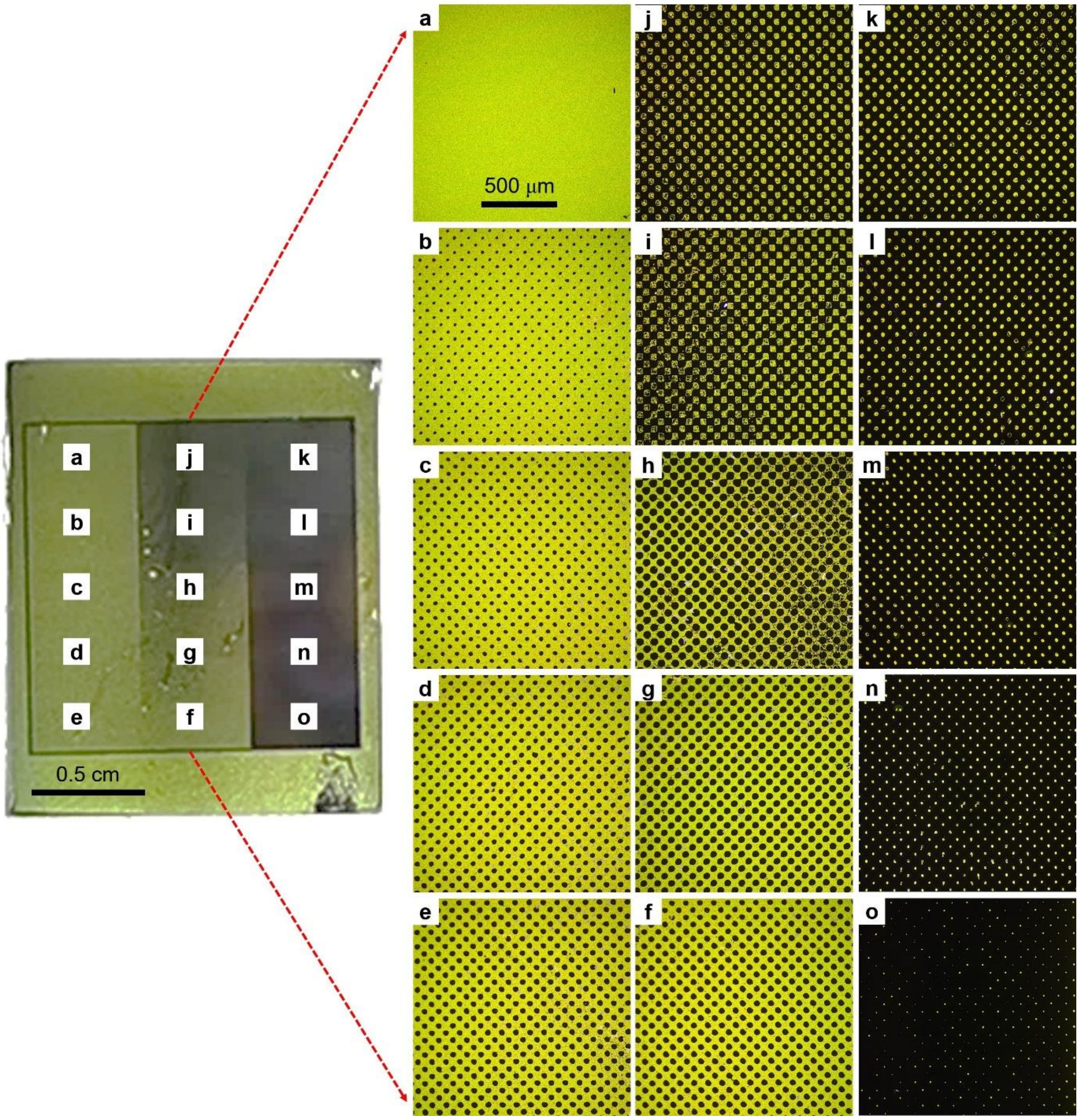

Supplementary Figure 24 Halftone dots with varying size or spacing made of patterned nanomesh are used to reproduce gradient image. To realize the above image patterning, the corresponding halftone patterns made on polyester-based films were fabricated and used as photomasks. This halftone reprographic technique relies on a basic optical illusion: when the halftone dots are in a microscopic level, the human eye interprets the patterned areas as if they were continuous tones.

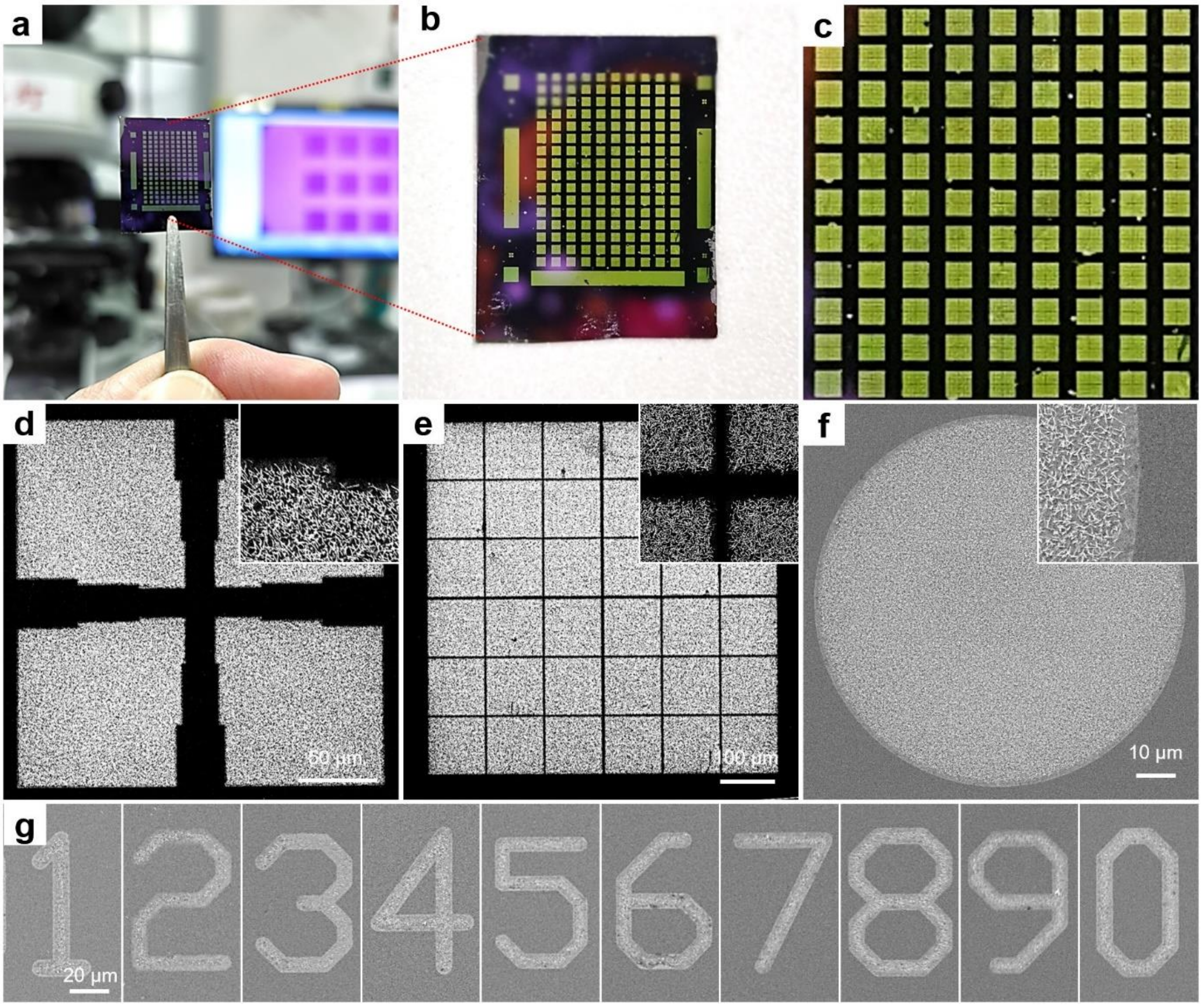

Supplementary Figure 25 (a-c) Photographs of patterned Te nanomesh on SiO_2/p^{++} Si substrates. (d-g) SEM images of patterned Te nanomesh with different shapes.

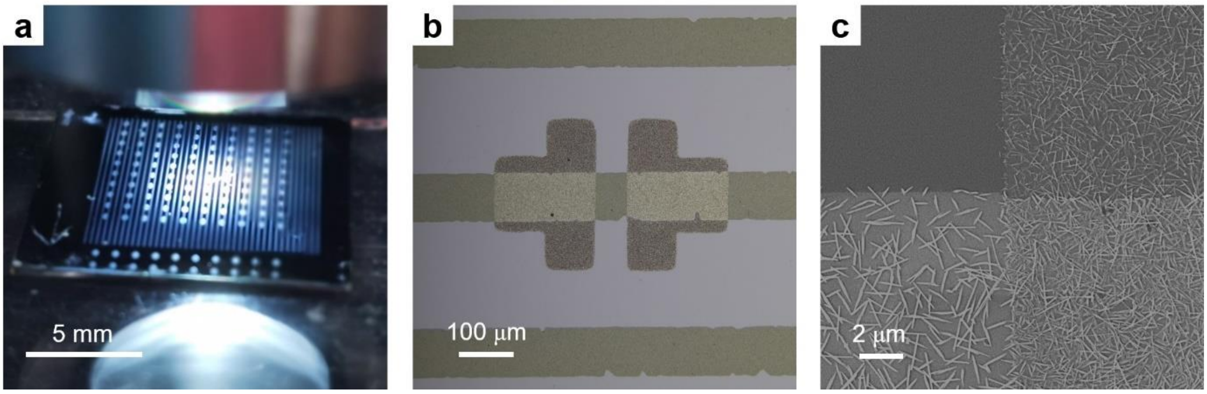

Supplementary Figure 26 (a) Photograph, (b) optical image, and (c) SEM image of all-nanomesh device. To fabricate all-nanomesh TFT on SiO_2 dielectric layer, the nanomesh growth process and the photolithography process were executed twice. The 3-hours grown Te nanomesh are used as the device channel, while the 5-hours grown conducting Te nanomesh are functioned as source/drain electrodes.

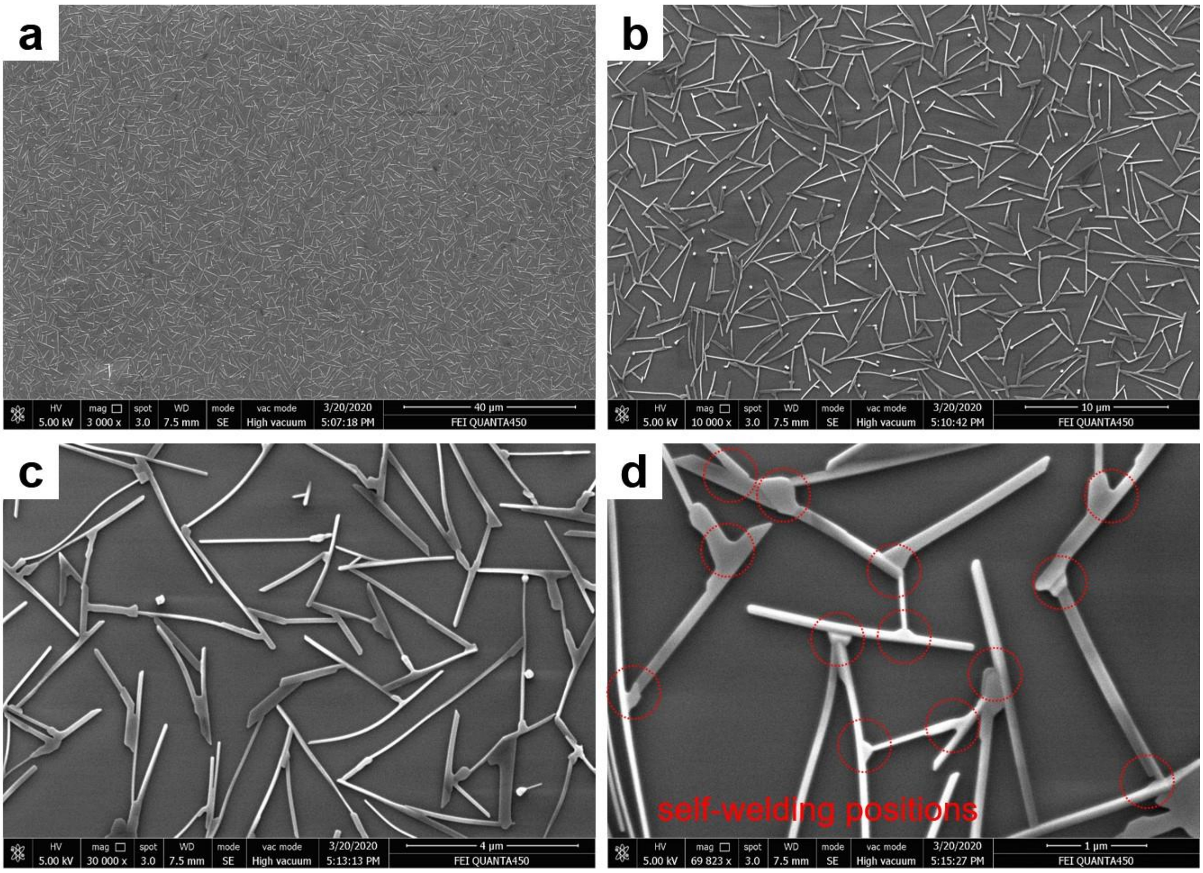

Supplementary Figure 27 (a-d) SEM images of Te nanomesh with a growth duration of 5 hours on SiO_2/p^{++} Si substrate. Compared to the 3-hours grown nanomesh depicted in Fig. 2b (main text), the increasing interconnection positions, the enlarging soldering points, and the thicker NW body are clearly observed with prolonging growth duration, showing the dramatically improved the welding status. All the morphological changes in nanomesh with longer growth duration would enhance the nanomesh conductivity as well as its mechanical strength.

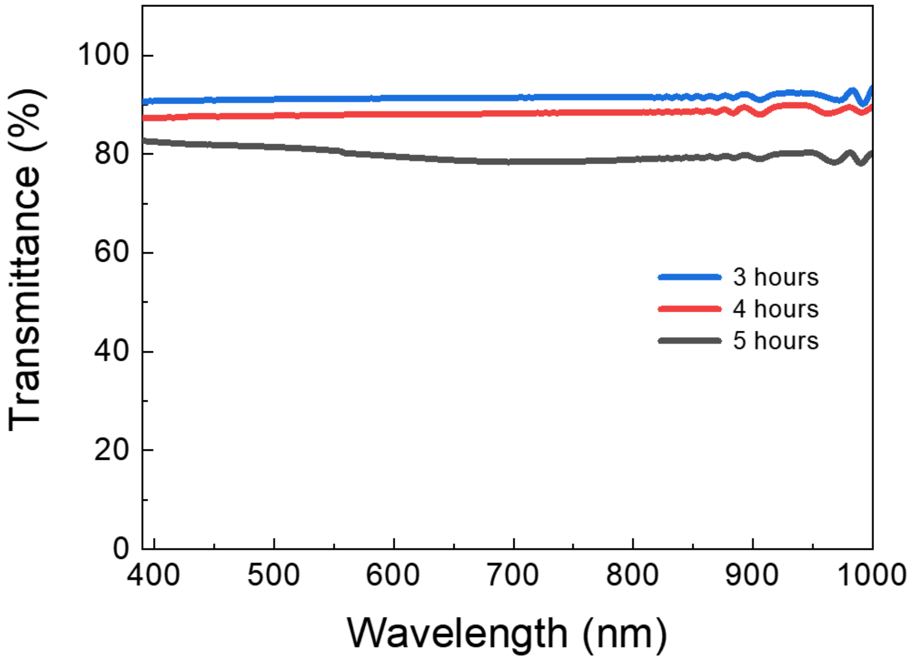

Supplementary Figure 28 Optical transmittance of Te nanomesh on glass with different growth durations. With increasing growth durations from 3 hours to 5 hours, the corresponding optical transmittances are decreased from ~ 90% to 80 % in the whole visible region. In specific, a high conductivity of 266 S cm^{-1} is obtained in Te nanomesh with a 5-hours growth time, while also holds good optical transparency above 80 %. This shows the potentials in applying Te nanomesh to p-type transparent conductors or device contact electrodes.

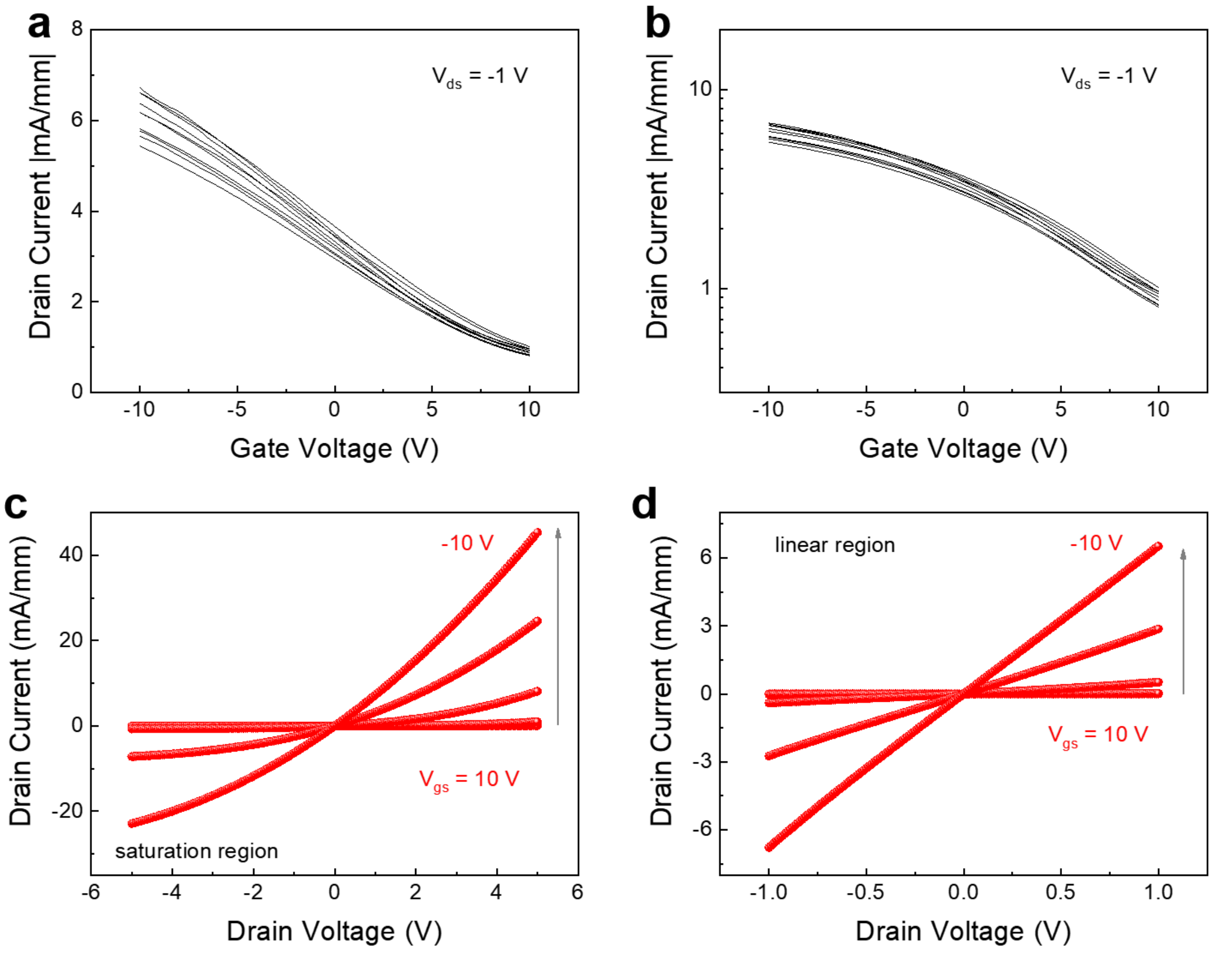

Supplementary Figure 29 Transfer curves of the 10 all-nanomesh TFTs using the (a) linear y-axis and (b) logarithmic y-axis. (c) Typical output curve containing saturation region of all-nanomesh TFT. The source-drain voltage sweeps from -5 V to 5 V. (d) Liner region of output curve containing saturation region of all-nanomesh TFT. The source-drain voltage sweeps from -1 V to 1 V. The typical asymmetric output curve, when applying positive and minus drain voltage, is because of the gate screening effect[14,15].

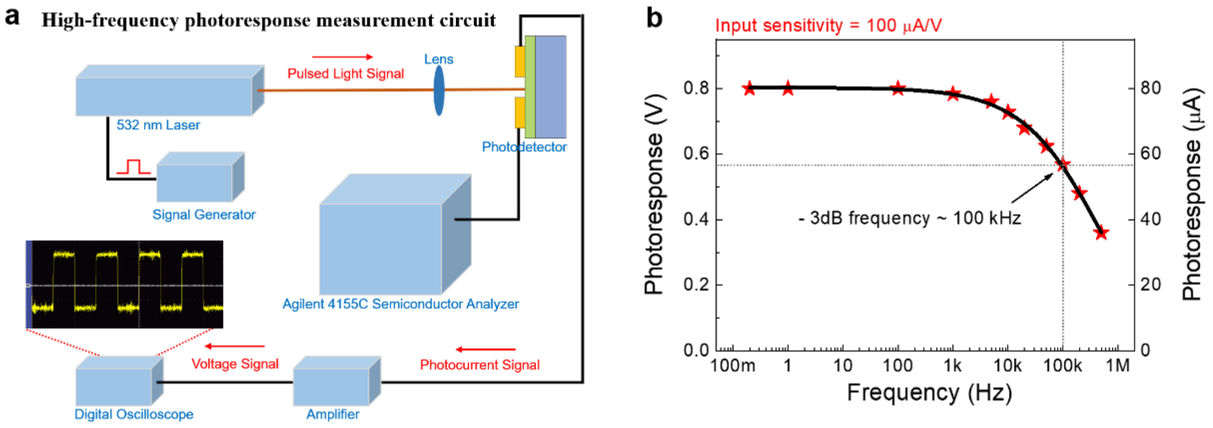

Supplementary Figure 30 (a) Experiment setup of high-frequency photoresponse measurement circuit. (b) Photoresponse (both output voltage photocurrent) as a function of frequency, showing 3 dB cutoff frequency of ~ 100 kHz.

Supplementary Note 8

To bypass the smallest signal interval time (> ms) collected by the semiconductor parameter analyzer (Agilent 4155C), we design a photoresponse measurement circuit (Figure S27). In specific, the light-induced current signals are first converted into voltage signals by a current amplifier, and then a digital oscilloscope is used to directly examined these dense voltage signals. In order to ensure the amplifier with maximum output voltage and minimal clipping/distortion, an appropriate input sensitivity of 100 μA/V is used to deliver voltage signals to digital oscilloscope. Therefore, the absolute current (I_a) could be simply estimated by multiplying output voltage (V_o) and input sensitivity (S_i). For instances, the peak photocurrent under a high-chopping frequency (e.g. 10 kHz) is calculated to be $I_a = V_o * S_i = 0.75\ V * 100\ \mu A/V = 72.8\ \mu A$, which is close to the static photocurrent of 80 μA measured at a low-chopping frequency (e.g. 0.2 Hz).

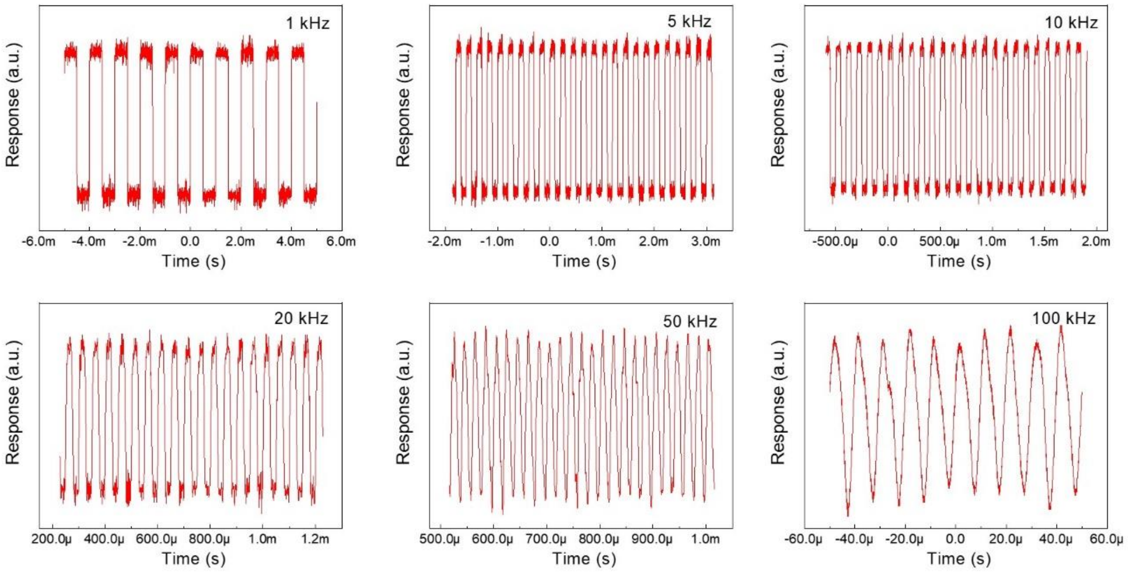

Supplementary Figure 31 Time-resolved photoresponse of paper-based Te nanomesh PDs with different chopped frequency from 1 kHz to 100 kHz. To measure the fast photoresponse of Te nanomesh, a photoresponse measurement circuit is built and utilized[16], in which the photocurrent signals are converted into voltage signals by a current amplifier and then examined by a digital oscilloscope. Anyway, the Te nanomesh devices exhibit excellent reliability and fast optical response in switching measurements with different chopping frequencies.

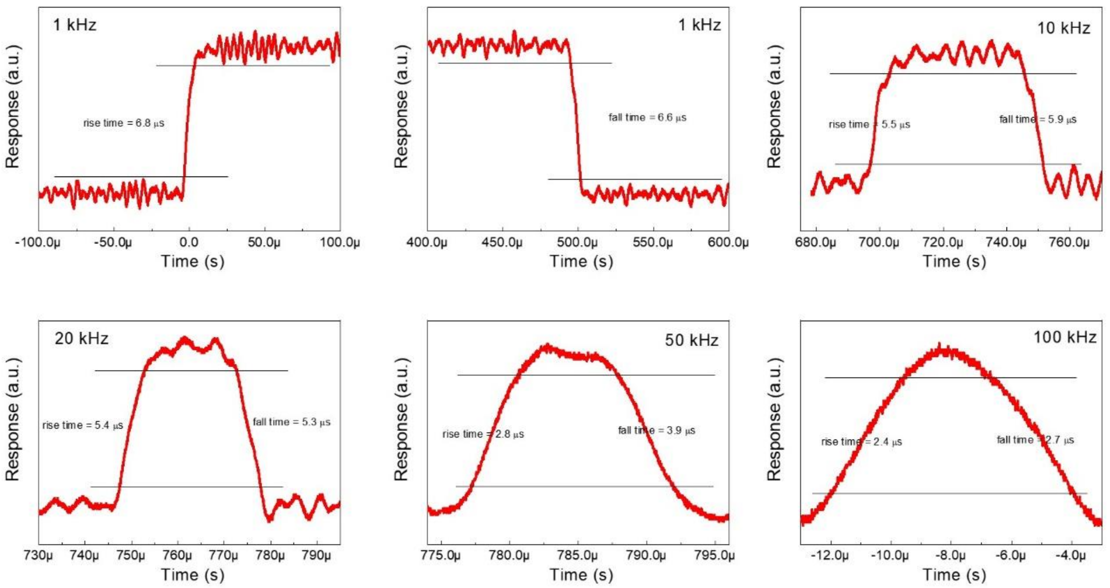

Supplementary Figure 32 Time-resolved photoresponse of paper-based Te nanomesh PDs with different chopped frequency from 1 kHz to 100 kHz. The response times, including the rise time and fall time, are determined as the time for the photocurrent to vary from 10% to 90%.

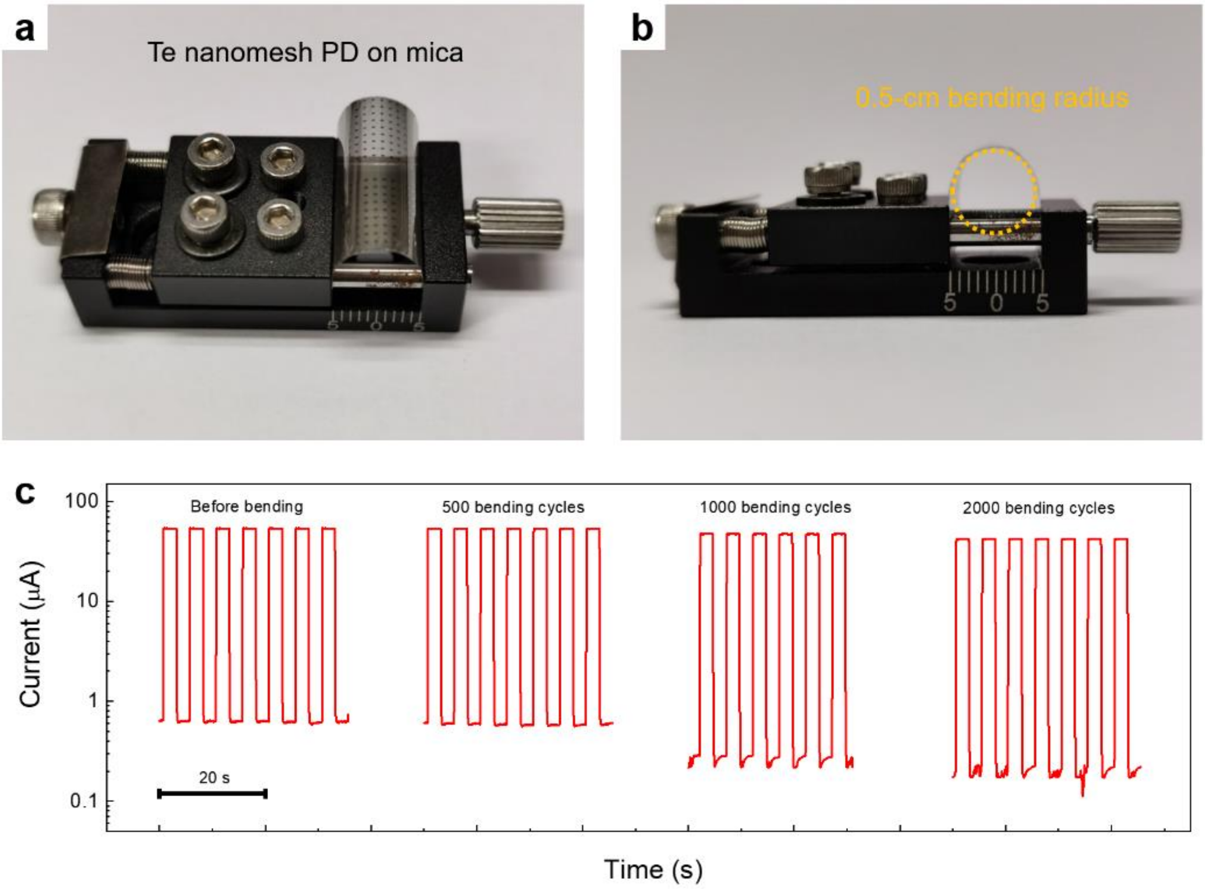

Supplementary Figure 33 (a, b) Photograph of Te nanomesh PD based on ultrathin mica substrate. (c) Photoresponse with varying bending cycles during the bending test from 0 to 2000 times.

Supplementary Note 9

The mica-based Te PD show a high photocurrent of 54 μA, measured at 1550 nm irradiation with an incident power density of 4 mW/cm^2, corresponding to a responsivity of 16.1 A/W. To determine the mechanical durability of nanomesh PDs, the on-off switching behavior of photocurrent was evaluated with different bending cycles at a fixed 0.5-cm bending radius (Supplementary Figure 33). No detectable photocurrent deterioration is observed through the entire bending test with bending cycles up to 2000 as presented in Supplementary Figure 33. Such robust device performance in the bending test is mainly driven by the intrinsic superior mechanical properties of self-welded Te nanomesh.

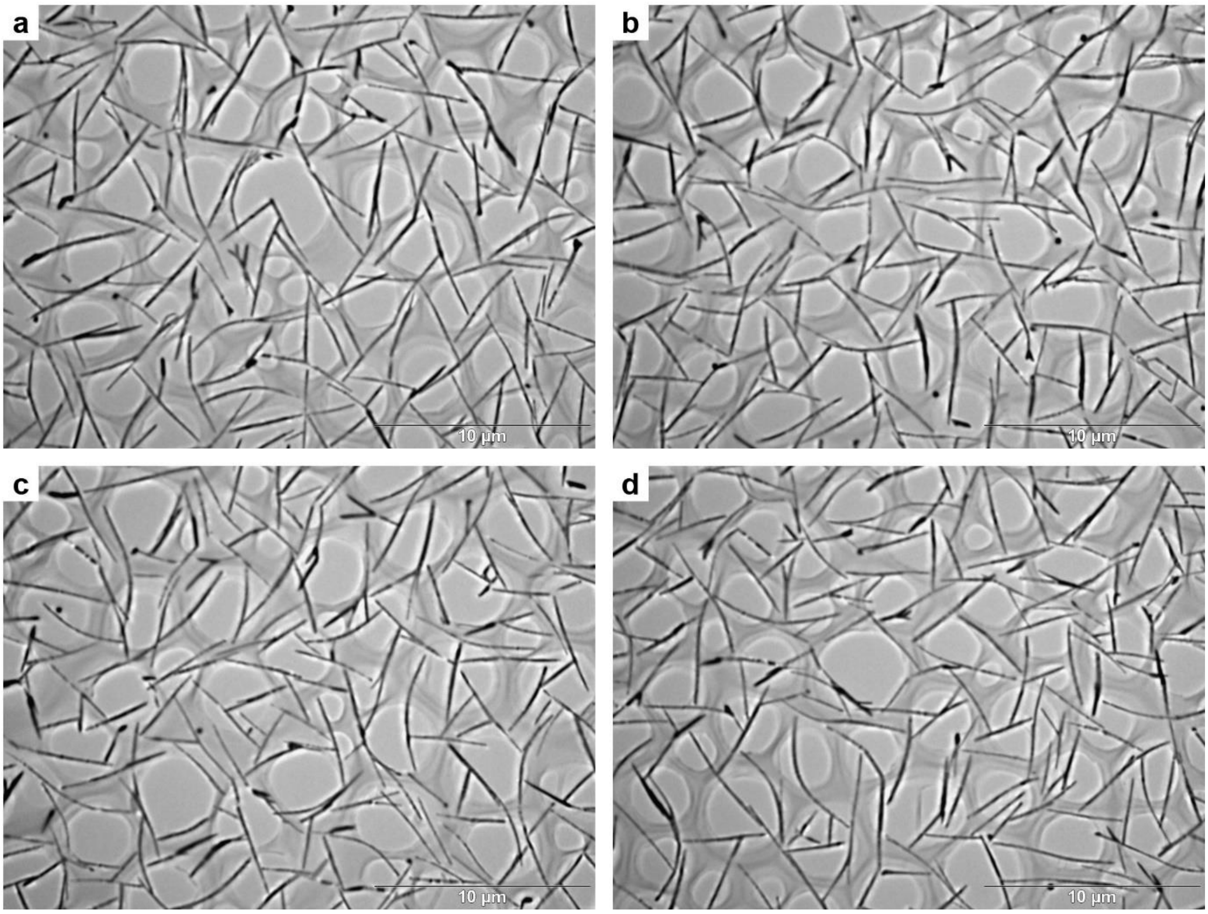

Supplementary Figure 34 (a-d) More TEM images of Te nanomesh/WS_2 monolayer mixed-dimensional heterostructures. The continuous Te nanomesh are clearly witnessed on the top surface of WS_2 layer.

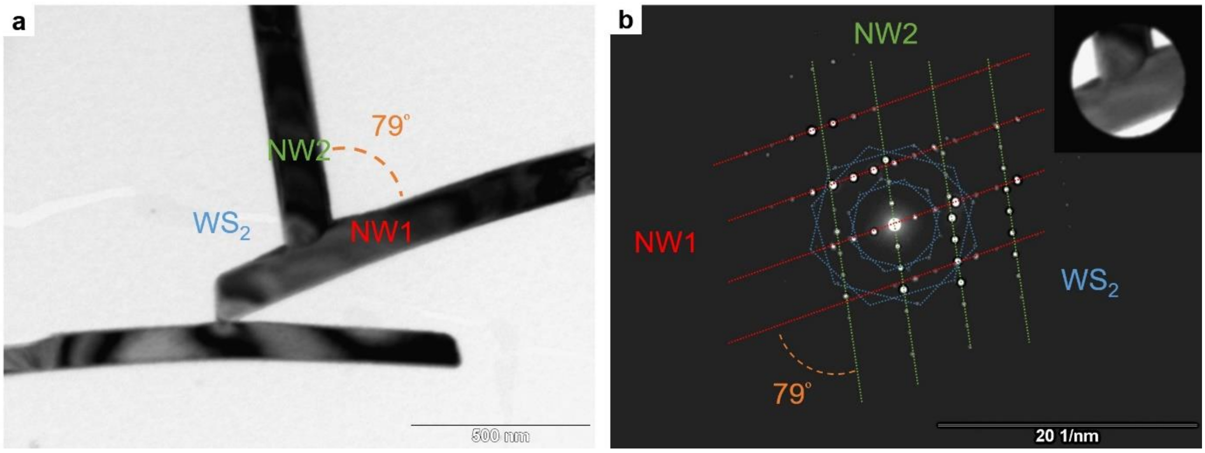

Supplementary Figure 35 (a) TEM image and (b) corresponding SAED pattern of Te nanomesh/WS_2 mixed-dimensional heterostructures. Three sets of diffraction spots are found in the SAED pattern collected from the Te nanomesh/WS_2 heterostructure, in which two sets belong to the welded Te NWs and one set belongs to the underlying WS_2 layer. It is noted that the angle between these two sets of diffraction spots (NW#1 and NW#2) is about 79°, being perfectly consistent with that in the TEM image.

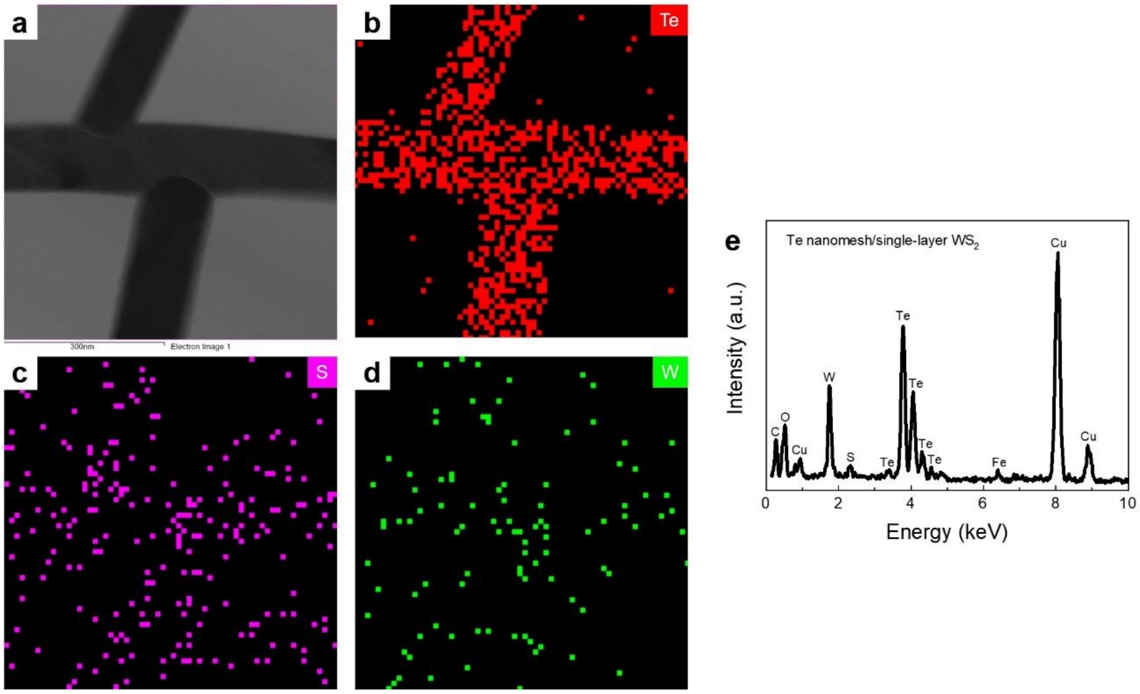

Supplementary Figure 36 (a) STEM image and (b) corresponding EDS mappings of Te nanomesh/WS_2 mixed-dimensional heterostructures. From EDS mapping, S is uniformly distributed throughout the entire heterostructure while Te is concentrated near the nanomesh, confirming the structural integrity of the mixed-dimensional heterostructure. (c) EDS spectrum of the Te nanomesh/WS_2 mixed-dimensional heterostructures.

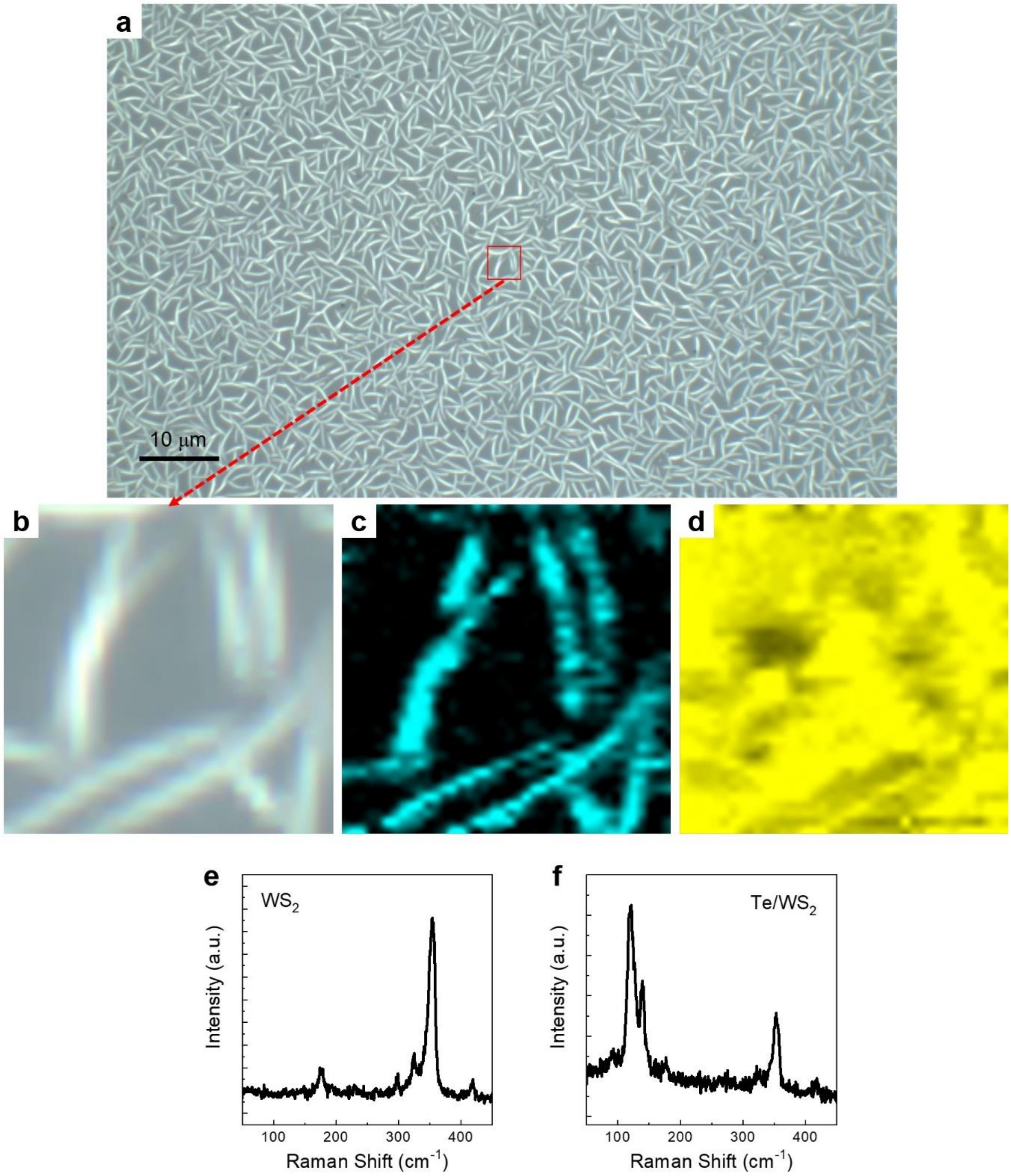

Supplementary Figure 37 (a, b) Optical images of Te nanomesh/monolayer WS_2 mixed-dimensional heterostructures grown on sapphire. (c, d) Raman mapping study of Te nanomesh/WS_2 heterostructures. From Raman mapping, the Te vibration mode is concentrated near the nanomesh, while the WS_2 vibration mode is uniformly distributed on the entire heterostructure, further designating the mixed-dimensional heterostructures. Raman spectrum of (e) WS_2 layer and (f) Te nanomesh/WS_2 heterostructures.

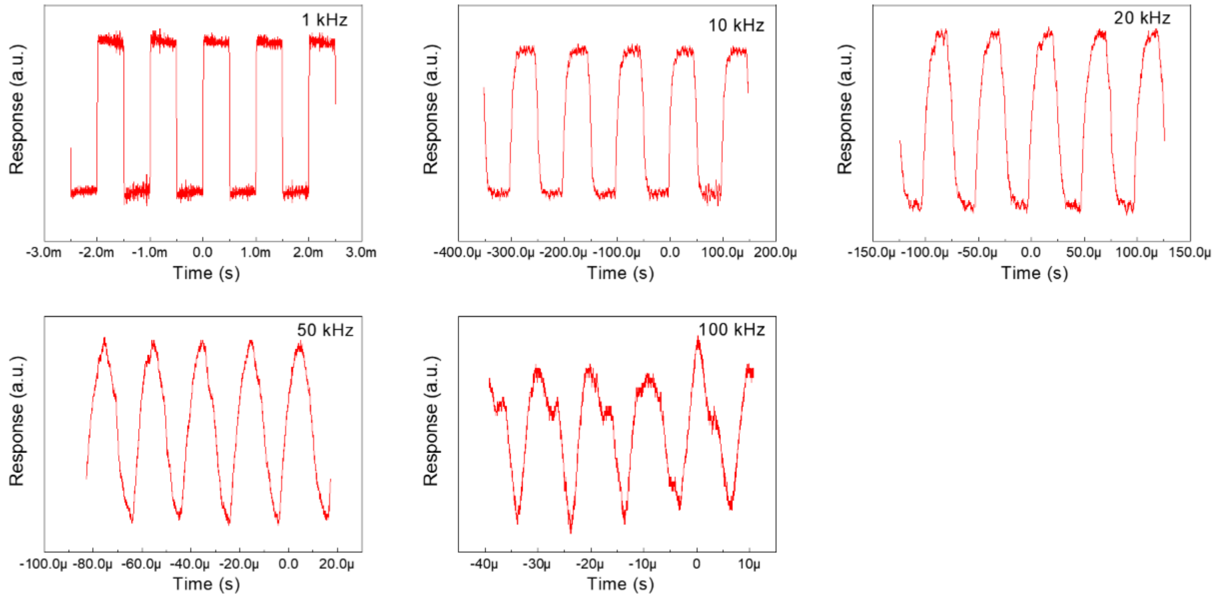

Supplementary Figure 38 Time-resolved photoresponse of the Te nanomesh/WS_2 monolayer mixed-dimensional PD with different chopping frequency from 1 kHz to 100 kHz.

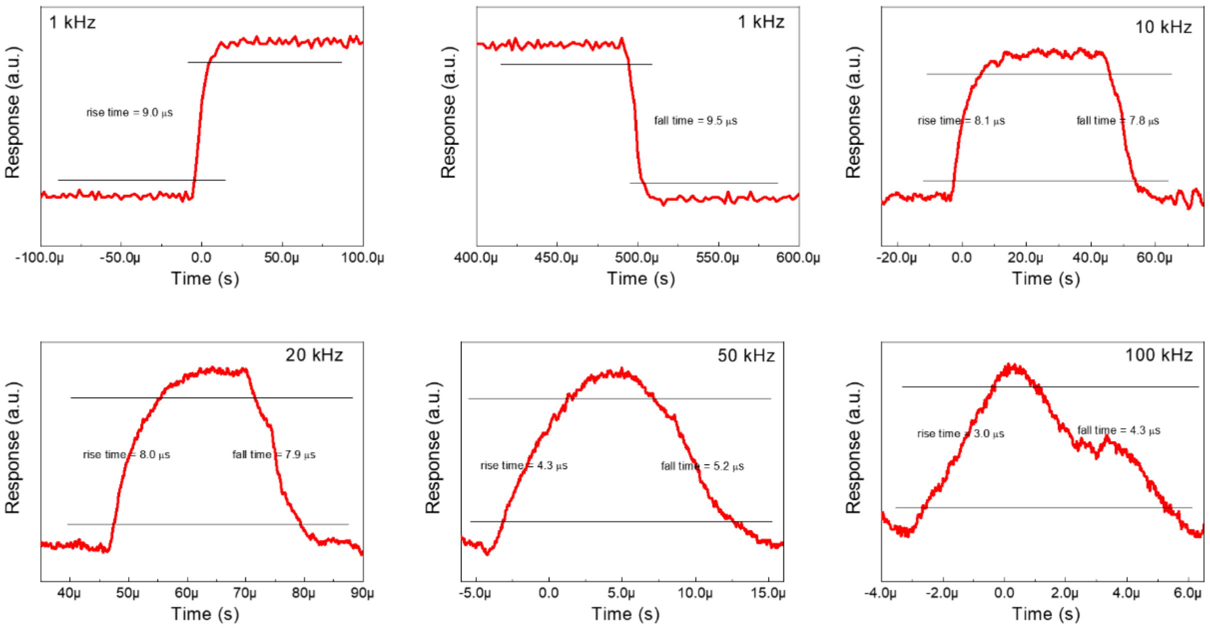

Supplementary Figure 39 Time-resolved photoresponse of the Te nanomesh/WS_2 monolayer mixed-dimensional PD with different chopping frequency from 1 kHz to 100 kHz. The response times, including the rise time and fall time, are determined as the time for the photocurrent to vary from 10% to 90%.

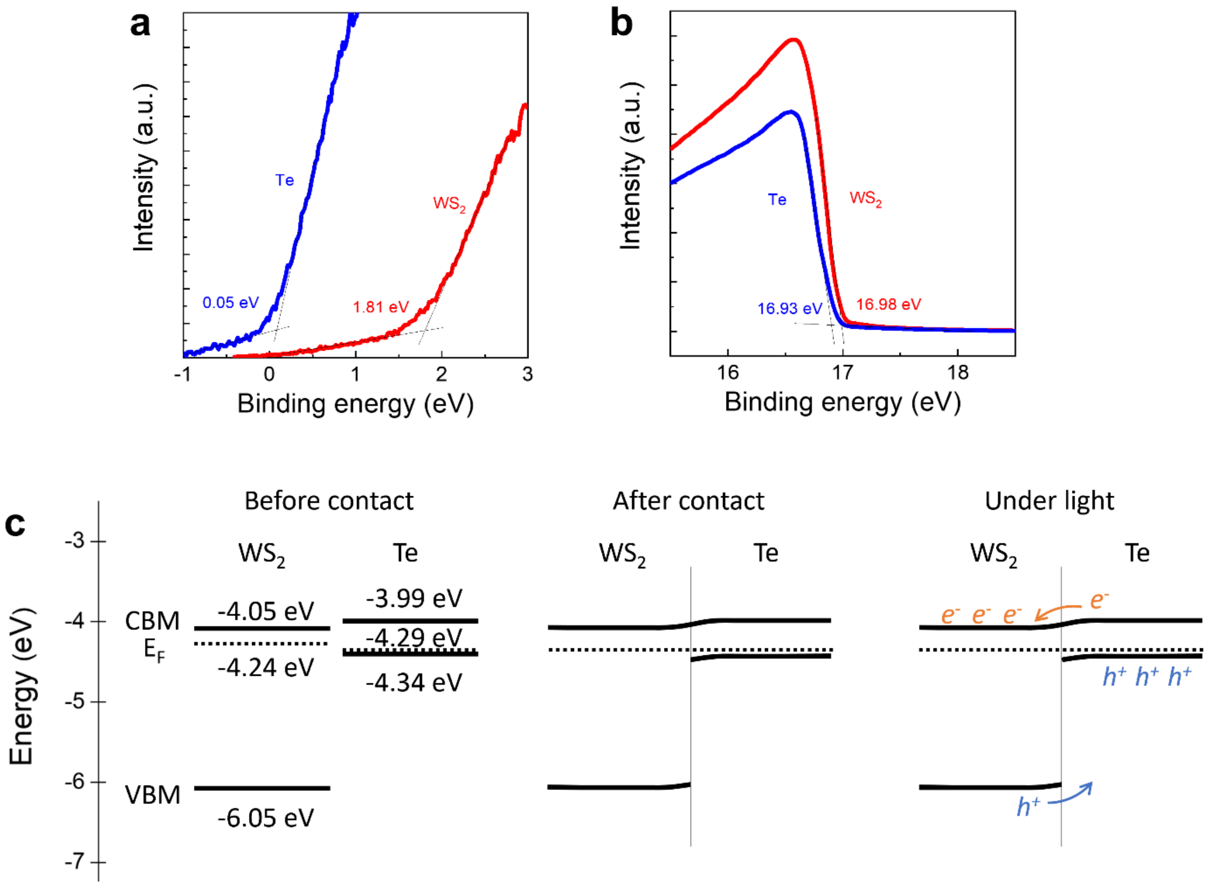

Supplementary Figure 40 UPS analysis for (a) valence-band regions and (b) secondary electron cutoff regions of Te nanomesh and WS_2. (c) Energy level diagram of the type-II band alignment in the Te nanomesh/WS_2 heterostructure before contact, after contact, and under light irradiation.

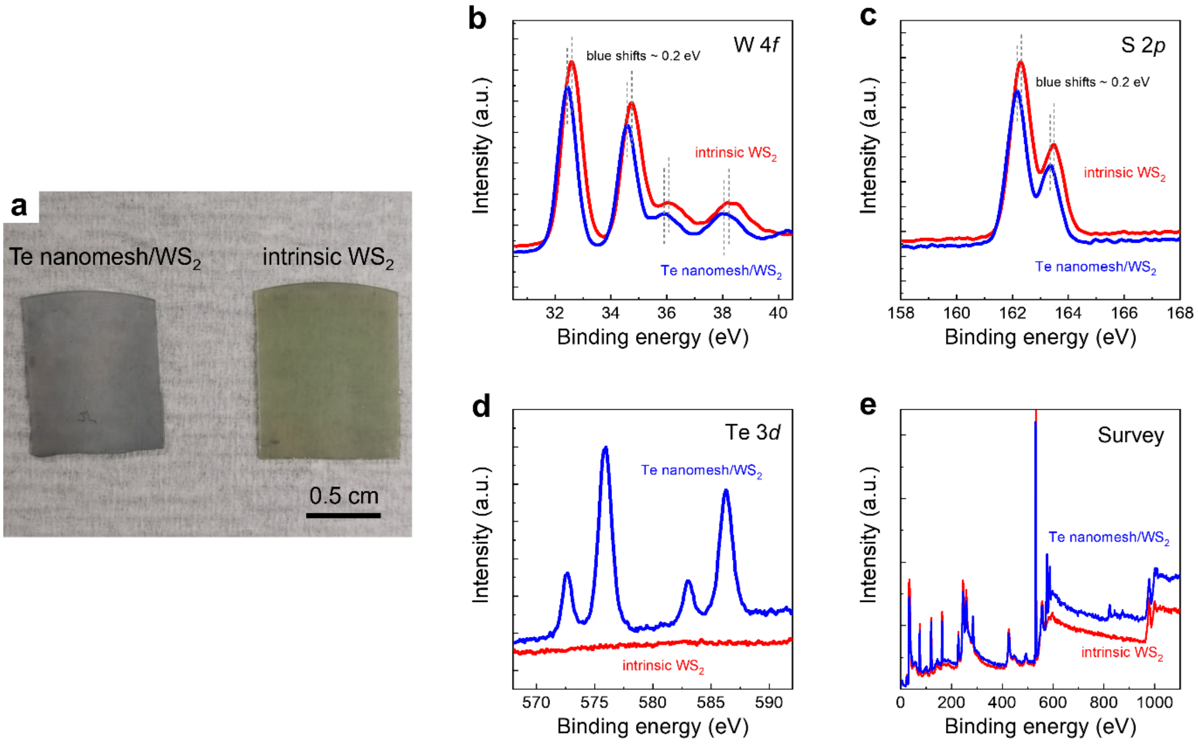

Supplementary Figure 41 (a) Photograph of the intrinsic WS_2 layer and Te nanomesh/WS_2 heterostructure. XPS spectra of (b) W 4*f*, (c) S 2*p*, (d) Te 3*d*, and (e) survey analysis of the intrinsic WS_2 layer and Te nanomesh/WS_2 heterostructure. The blue shifts (~ 0.2 eV) of W 4*f* peaks and S 2*p* peaks after Te nanomesh deposition confirms the charge transfer doping in WS_2 layer.

Supplementary Table 1 The surface energies of different substrates used in this work.

Growth surfaces	Surface energy [mJ m^{-2}]	Reference
**SiO_2**	287	[17]
**mica**	170	[18]
**sapphire**	72.8	[19]
**BN**	55.78	[20]
**WS_2**	46.5	[21]
**PI**	44	[22]
**paper**	47.2	[23]
**photoresist**	30	[24]
**PDMS**	19	[25]

Supplementary Table 2 The coefficient of friction of different vdWs interfaces.

vdWs interfaces	Coefficient of friction	Reference
**BN/Gr**	1×10^{-4}	[26]
**MoS_2/SiO_2**	$1.3 \pm 0.5 \times10^{-4}$	[27]
**Gr/Gr**	3×10^{-4}	[26]
**ReS_2/Gr**	9×10^{-4}	[26]
**Gr//SiO_2**	1×10^{-3}	[28]
**TaS_2/Gr**	1.1×10^{-3}	[26]
**MoS_2/Gr**	1.8×10^{-3}	[26]
**Gr/DLC**	4×10^{-3}	[29]
**Te NW/SiO_2**	$3.7 \pm 1.5 \times10^{-4}$	This work

Notes: Gr = graphene; DLC = diamondlike carbon.

Supplementary Table 3 Performance summary of TFTs based on p-type semiconducting thin films including metal oxides, metal halides, perovskites, organic materials, CNT thin films, and Te films.

Thin-film materials		Fabrication temperature	Hole mobility [cm^2/Vs]	Year	Reference
**Metal oxides**	SnO	450 °C	0.13	2012	[30]
	Cu_2O	700 °C	0.16	2013	[31]
	Cu_2O	600 °C	0.29	2015	[32]
	CuO	250 °C	0.30	2017	[33]
	CuO	220 °C	0.15	2019	[34]
	NiO	250 °C	0.07	2016	[35]
	NiO	250 °C	0.48	2019	[36]
**Metal halides**	CuI	60 °C	1.86	2018	[37]
	CuI	RT	0.40	2018	[38]
	CuSnI	140 °C	9	2018	[39]
	CuBr	RT	0.15	2019	[40]
	CuI:Zn	80 °C	5.3	2020	[41]
**Perovskites halides**	$MAPbI_3$	85 °C	2.1	2016	[42]
	$MAPbI_3$	120 °C	4	2017	[43]
	$MAPbBr_3$	270 °C	3.6	2018	[44]
	$MAPbCl_3$	270 °C	3.8	2018	[44]
	$(PEA)_2SnI_4$	90 °C	15	2016	[45]
	$(PEA)_2SnI_4$	100 °C	3.5	2020	[46]
**Organic materials**	TIPS-pentacene	90 °C	4.6	2011	[47]
	C8-BTBT	100 °C	10	2014	[48]
	C10-DNTT	80 °C	5.8	2016	[49]
	C10-DNBDT	135 °C	7.2	2017	[50]
	DPh-DNTT	90 °C	4.3	2020	[51]
**CNTs**	CNT thin films	200 °C	50	2012	[52]
	CNT thin films	150 °C	9	2013	[53]
	CNT thin films	125 °C	3.92	2016	[54]
	CNT thin films	80 °C	12	2018	[55]
	CNT thin films	140 °C	14	2020	[56]
**Tellurium**	Te thin films	minus 80 °C	35	2019	[57]
	Te thin films	minus 80 °C	46	2020	[58]
	Te thin films	minus 80 °C	50	2020	[59]
**Te vdWs nanomesh**		100 °C	145	2021	This work

Supplementary Table 4 Performance summary of PDs based on tellurium, perovskite halides, metal oxides, group III-Vs, layered materials, and Te nanomesh.

Materials		R [A/W]	Rise time [µs]	Fall time [µs]	Year	Reference
**Tellurium**	Te nanoplate	162.4	4.4×10^{6}	2.8×10^{6}	2014	[60]
	Te nanosheet	-	1.09×10^{6}	2.51×10^{6}	2018	[61]
	Te@Se NW	1×10^{-4}	9×10^{4}	1×10^{5}	2019	[62]
	Te nanoflake	15	5×10^{5}	1×10^{6}	2019	[63]
	Te nanomesh	~2.5	3×10^{5}	6×10^{5}	2019	[64]
	Te nanobelt	254.2	510	300	2019	[65]
	Te nanoflake	1360	48.7	62.7	2020	[66]
**Perovskite halides**	$CsPbBr_3$	0.64	19	24	2016	[67]
	$CsPbBr_3$	0.18	1800	1000	2016	[68]
	$MAPbI_3$	13.5	80	240	2016	[69]
	$MAPbI_3$	4.95	100	100	2016	[70]
	$CsPbBr_3$	34	600	900	2017	[71]
	$CsPbBr_3$	31.1	16	380	2017	[72]
	$MAPbI_3$	410	220	790	2018	[73]
	$CsPbCl_3$	1183	5×10^{4}	5×10^{4}	2019	[74]
	$FA_{1-x}Cs_xPbI_3$	5.7	0.045	0.091	2017	[75]
	$MAPbBr_3$	0.0136	0.52	2.435	2020	[76]
**Metal oxides**	InGaZnO	1.95×10^{6}	9.3×10^{5}	2×10^{5}	2019	[77]
	Ga_2O_3	547	1×10^{6}	6×10^{5}	2012	[78]
	Ga_2O_3	3000	1×10^{5}	3×10^{4}	2019	[79]
	CuO	15.3	6.8×10^{5}	1.77×10^{6}	2019	[80]
	$ZnGa_2O_4$	474	4×10^{5}	7×10^{5}	2020	[81]
	ZnO	6830	3×10^{5}	3.1×10^{5}	2019	[82]
	ZnSnO	11.3	1.78×10^{6}	3.02×10^{6}	2021	[83]
**Group III-Vs**	InGaSb	1520	13	16	2020	[84]
	InGaSb	6000	38	53	2019	[85]
	InAs	-	1.2×10^{4}	6×10^{3}	2014	[86]
	InGaAs	7300	4.8×10^{5}	8.1×10^{5}	2020	[87]
	GaSb	61	1.95×10^{5}	3.8×10^{5}	2019	[88]
	InSb	311.5	4.2×10^{6}	5.5×10^{6}	2020	[89]
	InAs	4400	1400	600	2020	[90]
**Layered materials**	WS_2	0.0032	1.6×10^{5}	1.7×10^{5}	2019	[91]
	$PdSe_2$	42.1	7.45×10^{4}	9.31×10^{4}	2019	[92]
	Bi_2O_2Se	6.5	2800	4600	2018	[93]
	$PtTe_2$	0.005	2.4	32	2020	[94]
	b-PAs	16	12.4	8.6	2017	[95]
	InSe	274	1.51×10^{4}	6.37×10^{4}	2020	[96]

MoS_2	12	10	20	2019	[97]
$MoTe_2$	0.0048	0.006	0.006	2017	[98]
InSe	0.4	0.2	0.2	2020	[99]
**Te vdWs nanomesh**	23.3	2.5	3.1	2021	This work

Supplementary References

1 Qin, J.-K. *et al.* Raman response and transport properties of tellurium atomic chains encapsulated in nanotubes. *Nat. Electron.* **3**, 141-147 (2020).

2 Sen, S. *et al.* Synthesis of tellurium nanostructures by physical vapor deposition and their growth mechanism. *J Crystal Growth* **8**, 238-242 (2008).

3 Gurarslan, A. *et al.* Surface-energy-assisted perfect transfer of centimeter-scale monolayer and few-layer MoS_2 films onto arbitrary substrates. *ACS Nano* **8**, 11522-11528 (2014).

4 Bürger, J.-C. *et al.* Analysis of the Growth of Laterally Aligned SnO_2 Nanowires by Thermodynamic Considerations and Experiments. *Crystal Growth & Design* **21**, 191-199, doi:10.1021/acs.cgd.0c00983 (2021).

5 Friedl, M. *et al.* Template-Assisted Scalable Nanowire Networks. *Nano Lett.* **18**, 2666-2671, doi:10.1021/acs.nanolett.8b00554 (2018).

6 Berweger, S. *et al.* Imaging carrier inhomogeneities in ambipolar tellurene field effect transistors. *Nano Lett.* **19**, 1289-1294 (2019).

7 Wang, Y. *et al.* Field-effect transistors made from solution-grown two-dimensional tellurene. *Nat. Electron.* **1**, 228-236, doi:10.1038/s41928-018-0058-4 (2018).

8 Chang, H.-M. *et al.* Germanium-doped metallic ohmic contacts in black phosphorus field-effect transistors with ultra-low contact resistance. *Scientific Reports* **7**, 1-9 (2017).

9 Du, Y., Liu, H., Deng, Y. & Ye, P. D. Device perspective for black phosphorus field-effect transistors: contact resistance, ambipolar behavior, and scaling. *ACS Nano* **8**, 10035-10042 (2014).

10 Si, M., Lin, Z., Charnas, A. & Peide, D. Y. Scaled atomic-layer-deposited indium oxide nanometer transistors with maximum drain current exceeding 2 A/mm at drain voltage of 0.7 V. *IEEE Electron Device Letters* **42**, 184-187 (2020).

11 Liu, H., Neal, A. T. & Ye, P. D. Channel length scaling of MoS_2 MOSFETs. *ACS Nano* **6**, 8563-8569 (2012).

12 Dasika, P. *et al.* Contact-Barrier Free, High Mobility, Dual-Gated Junctionless Transistor Using Tellurium Nanowire. *Adv. Funct. Mater.* **31**, 2006278 (2021).

13 Qiao, J. *et al.* Few-layer Tellurium: one-dimensional-like layered elementary semiconductor with striking physical properties. *Science Bulletin* **63**, 159-168 (2018).

14 See, G. H. *et al.* A compact model satisfying Gummel symmetry in higher order derivatives and applicable to asymmetric MOSFETs. *IEEE Trans. Electron Devices* **55**, 624-631 (2008).

15 McAndrew, C. C. Validation of MOSFET model source–drain symmetry. *IEEE Tansactions on Electron Devices* **53**, 2202-2206 (2006).

16 Meng, Y. *et al.* Perovskite Core–Shell Nanowire Transistors: Interfacial Transfer Doping and Surface Passivation. *ACS Nano* **14**, 12749-12760 (2020).

17 Kinloch, A. J. *Adhesion and adhesives: science and technology*. (Springer Science & Business Media,

1987).

18 Christenson, H. Adhesion and surface energy of mica in air and water. *The Journal of Physical Chemistry* **97**, 12034-12041 (1993).

19 Kempiński, M., Jenczyk, J., Jurga, S. & Śliwińska-Bartkowiak, M. Wetting of nanostructurized sapphire and gold surfaces. *Acta Phys. Pol., A* **132**, 185-188 (2017).

20 Hyun, C. *Investigation of wetting and surface energy of hexagonal boron nitride*, UNIST, (2018).

21 Gaur, A. P. *et al.* Surface energy engineering for tunable wettability through controlled synthesis of MoS_2. *Nano Lett.* **14**, 4314-4321 (2014).

22 Mittal, K. L. *Polymer surface modification: relevance to adhesion*. Vol. 3 (CRC Press, 2004).

23 El Omari, H., Ablouh, E.-h., Brouillette, F., Taourirte, M. & Belfkira, A. New method for determining paper surface energy per contact angle. *Cellulose* **26**, 9295-9309 (2019).

24 Hu, W., Yang, B., Peng, C. & Pang, S. Three-dimensional SU-8 structures by reversal UV imprint. *Journal of Vacuum Science Technology B* **24**, 2225-2229 (2006).

25 Vudayagiri, S., Junker, M. D. & Skov, A. L. Factors affecting the surface and release properties of thin polydimethylsiloxane films. *Polym. J.* **45**, 871-878 (2013).

26 Liu, Y. *et al.* Interlayer friction and superlubricity in single-crystalline contact enabled by two-dimensional flake-wrapped atomic force microscope tips. *ACS Nano* **12**, 7638-7646 (2018).

27 Li, H. *et al.* Superlubricity between MoS_2 monolayers. *Adv. Mater.* **29**, 1701474 (2017).

28 Zhang, S. *et al.* Tuning friction to a superlubric state via in-plane straining. *Proceedings of the National Academy of Sciences* **116**, 24452-24456 (2019).

29 Berman, D., Deshmukh, S. A., Sankaranarayanan, S. K., Erdemir, A. & Sumant, A. V. Macroscale superlubricity enabled by graphene nanoscroll formation. *Science* **348**, 1118-1122 (2015).

30 Okamura, K., Nasr, B., Brand, R. A. & Hahn, H. Solution-processed oxide semiconductor SnO in p-channel thin-film transistors. *J. Mater. Chem.* **22**, 4607-4610 (2012).

31 Kim, S. Y. *et al.* p-Channel oxide thin film transistors using solution-processed copper oxide. *ACS applied materials interfaces* **5**, 2417-2421 (2013).

32 Yu, J. *et al.* Solution-processed p-type copper oxide thin-film transistors fabricated by using a one-step vacuum annealing technique. *Journal of Materials Chemistry C* **3**, 9509-9513 (2015).

33 Liu, A. *et al.* In situ one-step synthesis of p-type copper oxide for low-temperature, solution-processed thin-film transistors. *Journal of Materials Chemistry C* **5**, 2524-2530 (2017).

34 Liu, A., Zhu, H. & Noh, Y.-Y. Polyol Reduction: A Low-Temperature Eco-Friendly Solution Process for p-Channel Copper Oxide-Based Transistors and Inverter Circuits. *ACS applied materials interfaces* **11**, 33157-33164 (2019).

35 Liu, A. *et al.* Hole mobility modulation of solution-processed nickel oxide thin-film transistor based on high-k dielectric. *Appl. Phys. Lett.* **108**, 233506 (2016).

36 Xu, W. *et al.* p-Type transparent amorphous oxide thin-film transistors using low-temperature solution-processed nickel oxide. *Journal of Alloys Compounds* **806**, 40-51 (2019).

37 Choi, C.-H. *et al.* Low-temperature, inkjet printed p-type copper (I) iodide thin film transistors. *Journal of Materials Chemistry C* **4**, 10309-10314 (2016).

38 Liu, A. *et al.* Room-Temperature Solution-Synthesized p-Type Copper (I) Iodide Semiconductors for Transparent Thin-Film Transistors and Complementary Electronics. *Adv. Mater.* **30**, 1802379 (2018).

39 Jun, T., Kim, J., Sasase, M. & Hosono, H. Material Design of p-Type Transparent Amorphous Semiconductor, Cu–Sn–I. *Adv. Mater.* **30**, 1706573 (2018).

40 Zhu, H., Liu, A. & Noh, Y.-Y. Transparent Inorganic Copper Bromide (CuBr) p-Channel Transistors Synthesized From Solution at Room Temperature. *IEEE Electron Device Letters* **40**, 769-772 (2019).

41 Liu, A. *et al.* High-performance p-channel transistors with transparent Zn doped-CuI. *Nat. Commun.* **11**, 4309 (2020).

42 Cho, N. *et al.* Pure crystal orientation and anisotropic charge transport in large-area hybrid perovskite films. *Nat. Commun.* **7**, 13407, doi:10.1038/ncomms13407 (2016).

43 Li, D. *et al.* The Effect of Thermal Annealing on Charge Transport in Organolead Halide Perovskite Microplate Field-Effect Transistors. *Adv. Mater.* **29**, 1601959, doi:10.1002/adma.201601959 (2017).

44 Yu, W. *et al.* Single crystal hybrid perovskite field-effect transistors. *Nat. Commun.* **9**, 5354, doi:10.1038/s41467-018-07706-9 (2018).

45 Matsushima, T. *et al.* Solution-Processed Organic-Inorganic Perovskite Field-Effect Transistors with High Hole Mobilities. *Adv. Mater.* **28**, 10275-10281, doi:10.1002/adma.201603126 (2016).

46 Zhu, H. *et al.* High-Performance and Reliable Lead-Free Layered-Perovskite Transistors. *Adv. Mater.* **32**, 2002717 (2020).

47 Giri, G. *et al.* Tuning charge transport in solution-sheared organic semiconductors using lattice strain. *Nature* **480**, 504-508 (2011).

48 He, D. *et al.* Two-dimensional quasi-freestanding molecular crystals for high-performance organic field-effect transistors. *Nat. Commun.* **5**, 1-7 (2014).

49 Uemura, T. *et al.* On the Extraction of Charge Carrier Mobility in High-Mobility Organic Transistors. *Adv. Mater.* **28**, 151-155 (2016).

50 Rolin, C. *et al.* Charge carrier mobility in thin films of organic semiconductors by the gated van der Pauw method. *Nat. Commun.* **8**, 1-9 (2017).

51 Borchert, J. W. *et al.* Flexible low-voltage high-frequency organic thin-film transistors. *Sci. Adv.* **6**, eaaz5156 (2020).

52 Wang, C. *et al.* Extremely bendable, high-performance integrated circuits using semiconducting carbon nanotube networks for digital, analog, and radio-frequency applications. *Nano Lett.* **12**, 1527-1533 (2012).

53 Lau, P. H. *et al.* Fully printed, high performance carbon nanotube thin-film transistors on flexible substrates. *Nano Lett.* **13**, 3864-3869 (2013).

54 Cao, X. *et al.* Fully screen-printed, large-area, and flexible active-matrix electrochromic displays using carbon nanotube thin-film transistors. *ACS Nano* **10**, 9816-9822 (2016).

55 Cardenas, J. A. *et al.* In-place printing of carbon nanotube transistors at low temperature. *ACS Applied Nano Materials* **1**, 1863-1869 (2018).

56 Zhang, Z., Du, C., Jiao, H. & Zhang, M. Polyvinyl Alcohol/SiO_2 Hybrid Dielectric for Transparent Flexible/Stretchable All-Carbon-Nanotube Thin-Film-Transistor Integration. *Advanced Electronic Materials* **6**, 1901133 (2020).

57 Zhao, C. *et al.* Evaporated tellurium thin films for p-type field-effect transistors and circuits. *Nat. Nanotechnol.* **15**, 53-58, doi:10.1038/s41565-019-0585-9 (2020).

58 Tan, C. *et al.* Evaporated Se_xTe_{1-x} Thin Films with Tunable Bandgaps for Short Wave Infrared Photodetectors. *Adv. Mater.*, 2001329, doi:10.1002/adma.202001329 (2020).

59 Zhao, C., Hurtado, L. & Javey, A. Thermal stability for Te-based devices. *Appl. Phys. Lett.* **117**, 192104 (2020).

60 Wang, Q. *et al.* Van der Waals epitaxy and photoresponse of hexagonal tellurium nanoplates on flexible mica sheets. *ACS Nano* **8**, 7497-7505 (2014).

61 Peng, J. *et al.* Two-Dimensional Tellurium Nanosheets Exhibiting an Anomalous Switchable Photoresponse with Thickness Dependence. *Angew. Chem. Int. Ed.* **57**, 13533-13537 (2018).
62 Huang, W. *et al.* Enhanced Photodetection Properties of Tellurium@ Selenium Roll-to-Roll Nanotube Heterojunctions. *Small* **15**, 1900902 (2019).
63 Zhang, X. *et al.* Hydrogen-Assisted Growth of Ultrathin Te Flakes with Giant Gate-Dependent Photoresponse. *Adv. Funct. Mater.* **29**, 1906585 (2019).
64 Dang, S. *et al.* Piezoelectric modulation of broadband photoresponse of flexible tellurium nanomesh photodetectors. *Nanotechnology* **31**, 095502 (2019).
65 Kang, S. *et al.* Broad spectral response of an individual tellurium nanobelt grown by molecular beam epitaxy. *Nanoscale* **11**, 1879-1886 (2019).
66 Tong, L. *et al.* Stable mid-infrared polarization imaging based on quasi-2D tellurium at room temperature. *Nat. Commun.* **11**, 2308 (2020).
67 Song, J. *et al.* Monolayer and Few-Layer All-Inorganic Perovskites as a New Family of Two-Dimensional Semiconductors for Printable Optoelectronic Devices. *Adv. Mater.* **28**, 4861-4869, doi:10.1002/adma.201600225 (2016).
68 Li, X. *et al.* Healing All-Inorganic Perovskite Films via Recyclable Dissolution-Recyrstallization for Compact and Smooth Carrier Channels of Optoelectronic Devices with High Stability. *Adv. Funct. Mater.* **26**, 5903-5912, doi:10.1002/adfm.201601571 (2016).
69 Deng, W. *et al.* Aligned Single-Crystalline Perovskite Microwire Arrays for High-Performance Flexible Image Sensors with Long-Term Stability. *Adv. Mater.* **28**, 2201-2208, doi:10.1002/adma.201505126 (2016).
70 Gao, L. *et al.* Passivated Single-Crystalline $CH_3NH_3PbI_3$ Nanowire Photodetector with High Detectivity and Polarization Sensitivity. *Nano Lett.* **16**, 7446-7454, doi:10.1021/acs.nanolett.6b03119 (2016).
71 Liu, X. *et al.* Low-Voltage Photodetectors with High Responsivity Based on Solution-Processed Micrometer-Scale All-Inorganic Perovskite Nanoplatelets. *Small* **13**, 1700364, doi:10.1002/smll.201700364 (2017).
72 Li, X. *et al.* Constructing Fast Carrier Tracks into Flexible Perovskite Photodetectors To Greatly Improve Responsivity. *ACS Nano* **11**, 2015-2023, doi:10.1021/acsnano.6b08194 (2017).
73 Zhou, Q. *et al.* Nanochannel-Assisted Perovskite Nanowires: From Growth Mechanisms to Photodetector Applications. *ACS Nano* **12**, 8406-8414, doi:10.1021/acsnano.8b03826 (2018).
74 Meng, Y. *et al.* Direct Vapor–Liquid–Solid Synthesis of All-Inorganic Perovskite Nanowires for High-Performance Electronics and Optoelectronics. *ACS Nano* **13**, 6060-6070, doi:10.1021/acsnano.9b02379 (2019).
75 Liang, F. X. *et al.* Broadband, ultrafast, self-driven photodetector based on Cs-doped $FAPbI_3$ perovskite thin film. *Adv. Opt. Mater.* **5**, 1700654 (2017).
76 Geng, X. *et al.* Ultrafast photodetector by integrating perovskite directly on silicon wafer. *ACS Nano* **14**, 2860-2868 (2020).
77 Li, F. *et al.* High-Performance Transparent Ultraviolet Photodetectors Based on InGaZnO Superlattice Nanowire Arrays. *ACS Nano* **13**, 12042-12051, doi:10.1021/acsnano.9b06311 (2019).
78 Tian, W. *et al.* In-doped Ga_2O_3 nanobelt based photodetector with high sensitivity and wide-range photoresponse. *J. Mater. Chem.* **22**, 17984-17991 (2012).
79 Qin, Y. *et al.* Enhancement-Mode beta-Ga_2O_3 Metal–Oxide–Semiconductor Field-Effect Solar-Blind Phototransistor With Ultrahigh Detectivity and Photo-to-Dark Current Ratio. *IEEE Electron Device*

Letters **40**, 742-745 (2019).
80 Song, H.-J. *et al.* High-performance copper oxide visible-light photodetector via grain-structure model. *Scientific reports* **9**, 7334 (2019).
81 Huang, P.-H. *et al.* Energy-Saving $ZnGa_2O_4$ Phototransistor Improved by Thermal Annealing. *ACS Applied Electronic Materials* **2**, 3515-3521 (2020).
82 Wang, Y. *et al.* High performance charge-transfer induced homojunction photodetector based on ultrathin ZnO nanosheet. *Appl. Phys. Lett.* **114**, 011103 (2019).
83 Huang, C.-Y. *et al.* High-performance solution-processed ZnSnO metal–semiconductor–metal ultraviolet photodetectors via ultraviolet/ozone photo-annealing. *Semiconductor Science Technology* **36**, 095013 (2021).
84 Li, D. *et al.* Flexible Near-Infrared InGaSb Nanowire Array Detectors with Ultrafast Photoconductive Response Below 20 μs. *Adv. Opt. Mater.* **8**, 2001201 (2020).
85 Li, D. *et al.* Ultra-fast photodetectors based on high-mobility indium gallium antimonide nanowires. *Nat. Commun.* **10**, 1664 (2019).
86 Guo, N. *et al.* Anomalous and highly efficient InAs nanowire phototransistors based on majority carrier transport at room temperature. *Adv. Mater.* **26**, 8203-8209 (2014).
87 Zhang, H. *et al.* Enhanced performance of near-infrared photodetectors based on InGaAs nanowires enabled by a two-step growth method. *Journal of Materials Chemistry C* **8**, 17025-17033 (2020).
88 Sun, J. *et al.* Ultrahigh hole mobility of Sn-catalyzed GaSb nanowires for high speed infrared photodetectors. *Nano Lett.* **19**, 5920-5929 (2019).
89 Zhang, S. *et al.* Highly sensitive InSb nanosheets infrared photodetector passivated by ferroelectric polymer. *Adv. Funct. Mater.* **30**, 2006156 (2020).
90 Zhang, X. *et al.* Surface-states-modulated high-performance InAs nanowire phototransistor. *The Journal of Physical Chemistry Letters* **11**, 6413-6419 (2020).
91 Lan, C. *et al.* Utilizing a NaOH promoter to achieve large single-domain monolayer WS_2 films via modified chemical vapor deposition. *ACS applied materials interfaces* **11**, 35238-35246 (2019).
92 Long, M. *et al.* Palladium diselenide long-wavelength infrared photodetector with high sensitivity and stability. *ACS Nano* **13**, 2511-2519 (2019).
93 Li, J. *et al.* High-performance near-infrared photodetector based on ultrathin Bi_2O_2Se nanosheets. *Adv. Funct. Mater.* **28**, 1706437 (2018).
94 Zeng, L. *et al.* Van der Waals epitaxial growth of mosaic-like 2D platinum ditelluride layers for room-temperature mid-infrared photodetection up to 10.6 μm. *Adv. Mater.* **32**, 2004412 (2020).
95 Amani, M., Regan, E., Bullock, J., Ahn, G. H. & Javey, A. Mid-wave infrared photoconductors based on black phosphorus-arsenic alloys. *ACS Nano* **11**, 11724-11731 (2017).
96 Curreli, N. *et al.* Liquid phase exfoliated indium selenide based highly sensitive photodetectors. *Adv. Funct. Mater.* **30**, 1908427 (2020).
97 Lv, L. *et al.* Reconfigurable two-dimensional optoelectronic devices enabled by local ferroelectric polarization. *Nat. Commun.* **10**, 1-10 (2019).
98 Bie, Y.-Q. *et al.* A $MoTe_2$-based light-emitting diode and photodetector for silicon photonic integrated circuits. *Nat. Nanotechnol.* **12**, 1124-1129 (2017).
99 Dai, M. *et al.* Ultrafast and sensitive self-powered photodetector featuring self-limited depletion region and fully depleted channel with van der Waals contacts. *ACS Nano* **14**, 9098-9106 (2020).
